# Comparative and pharmacological investigation of bEVs from eight *Lactobacillales* strains

**DOI:** 10.1038/s41598-025-12873-z

**Published:** 2025-07-26

**Authors:** Seoah Park, Jongsoo Mok, Hye-Min Yu, Hye-Jin An, Ga-Hyun Choi, Yeon-Seon Lee, Ki-Jin Kwon, Sung-Jun Choi, Kyung-Hee Kim, Soo-Jin Kim, Joonghoon Park

**Affiliations:** 1 Graduate School of International Agricultural Technology, Seoul National University, Seoul, Korea; 2 Institute of Green Bio Science & Technology, Seoul National University, Seoul, Korea; 3 Schofield Biome Research Lab, HK inno. N, Koreasu, Seoul, Korea; 4 Proteomics Core Facility, Research Core Center, Research Institute, National Cancer Center, Seoul, Korea

**Keywords:** bEV, Therapeutic indications, Skin health, Collagen, bEV-host interaction, Nanoparticles, Bacterial secretion, Pharmacology

## Abstract

Bacterial extracellular vesicles (bEVs) have therapeutic potential by mimicking the effects of the microbiome. Here, we characterized bEVs from eight gram-positive *Lactobacillales* strains, evaluating their therapeutic potential. In primary characterization, *Lactobacillus paracasei* produced the largest bEVs (82.5 nm), while *Lactococcus lactis* yielded the highest number (3.2 × 10⁹ particles/mL). *Lactobacillus plantarum* had the highest protein content (0.124 pg/particle), while *Lactobacillus salivarius* had the greatest lipid content (16.3 µg/particle). Lipid content significantly influenced cytotoxicity in HEK293T cells (*r*² = 0.366, *p* = 0.037). Connectivity Map (CMap) analysis revealed correlations between bEVs from *Lactobacillus rhamnosus*, *Lactobacillus fermentum*, *Lactobacillus acidophilus*, and *Streptococcus thermophilus* and approved drugs for skin health. Experimentally, these bEVs enhanced collagen synthesis in fibroblasts by up to 1.25-fold (*p* < 0.001). Proteomic analysis identified distinct protein sets for each bEV. Further analysis demonstrated interaction networks between bEV proteins and human proteins that promote collagen production through the JAK-STAT, PI3K-AKT, and focal adhesion pathways. In conclusion, this study highlights the strain-specific characteristics and therapeutic potential of bEVs in promoting collagen production, presenting a novel approach to discovering new indications for bEVs in potential skin care applications.

**Supplementary Information:**

The online version contains supplementary material available at 10.1038/s41598-025-12873-z.

## Introduction

Cell therapy involves the direct administration of cells derived from various sources to a patient to replace or repair damaged or dysfunctional cells in the body^1^. The mechanism of action of cell therapy may involve a paracrine effect, in which the administered cells interact with the tissues of the recipient to exert a therapeutic effect rather than direct regeneration of damaged cells^2^. Recent studies have revealed the role of the secretome of cells in the therapeutic effects of cell therapy^3^. The secretome contains a variety of proteins, growth factors, and signaling molecules that can regulate cell behavior and promote tissue regeneration^4^. Extracellular vesicles (EVs) constitute one type of secretome and are emerging as active ingredients responsible for many of the therapeutic effects of cell therapy. EVs, which are small lipid bilayer vesicles released from cells, act as vehicles to deliver bioactive molecules such as proteins, nucleic acids, and lipids to recipient cells and are known to influence gene expression and cell signaling and ultimately induce therapeutic effects^5–7^.

The microbiome is a community of microorganisms that reside in various parts of the body; these microorganisms interact closely with the host and play important roles in human health^8–10^. In this symbiotic relationship, the host provides nutrients and an appropriate environment in exchange for immunologic and metabolic benefits^11^. As a result, changes in the microbiome can contribute to the development of diseases, including immune-mediated, neurological, cardiovascular, and metabolic diseases^12–15^. Recent research has emphasized the role of microbe-derived EVs in mediating the effects of the microbiome on the host^16^. These EVs act as vesicles to deliver microbial molecules to host cells, enabling direct interkingdom interactions and signal transduction. The research on the specific cargo and mechanisms of action of microbial-derived EVs is a rapidly evolving, and this research may provide insights into the development of targeted interventions to manipulate the microbiome and improve human health^17^.

bEVs are formed through diverse mechanisms, including non-lytic membrane blebbing and explosive cell lysis, depending on the bacterial species and conditions^18,19^. In gram-positive bacteria, vesiculation may involve enzymatic remodeling of the thick peptidoglycan layer by autolysins or penicillin-binding proteins, facilitating the outward protrusion of the cytoplasmic membrane driven by turgor pressure^20^. These processes can occur constitutively or be triggered by environmental or genetic stimuli^21^. bEV secretion can increase in response to environmental cues, such as stressors or interactions with host cells. This inducible secretion allows bacteria to modulate the composition of bEV cargo in response to specific environmental conditions, potentially enhancing their survival or influencing host‒microbe interactions^22^.

bEVs possess several unique properties that make them attractive for mimicking the effects of the microbiome. First, they encapsulate various bioactive molecules, including proteins, lipids, nucleic acids, and metabolites, which can modulate host cellular processes. These molecules can be delivered to target cells, enabling communication and influencing host physiology^23^. Second, bEVs are relatively stable and resistant to degradation, allowing them to survive in various body fluids and potentially reach distant sites of action^24^. Third, bEVs can be engineered or modified to enhance their cargo delivery or target specific cell types, making them versatile tools for therapeutic interventions^25^. Pharmacological studies using bEVs have demonstrated promising results in animal and human studies. For example, in mice, bEVs alleviate symptoms of inflammatory bowel disease, enhance vaccine responses^26,27^and show potential as immune modulators^28^. These findings suggest that bEVs have the potential to mimic the effects of the microbiome and offer therapeutic benefits^29^. Despite the diverse pharmacological potential of bEVs, the number of bacterial strains from which bEVs have been isolated and studied is limited. Recent studies have provided valuable insights into bEVs derived from *Lactobacillales* species, including *Lactobacillus plantarum* (*L. plantarum*), *Lactobacillus fermentum* (*L. fermentum*), and *Lactobacillus gasseri (L. gasseri*), highlighting their distinct proteomic profiles and selective cargo packaging^20,30^. However, a comprehensive comparative evaluation of bEVs across multiple strains and their mechanisms of action is lacking^31,32^.

In this study, bEVs were isolated and characterized from eight gram-positive bacterial strains of the order *Lactobacillales*, and bEV-induced transcriptomic responses in human embryonic kidney 293T (HEK293T) cells were used to identify potential indications. We experimentally validated the effects of selected bEVs on collagen production using mouse embryonic fibroblasts (NIH3T3) and human hepatic stellate (LX-2) cells, and performed an integrated analysis of the bEV proteome, human proteins that interact with bEV proteins, and bEV-affected signaling molecules in humans. These comprehensive analyses allowed the mapping of signaling networks and identification of potential active ingredients in bEVs, providing valuable insights into the molecular mechanisms of bEVs in skin health.

## Results

### Characterization of bEVs from eight *Lactobacillales* strains

**Fig. 1:**
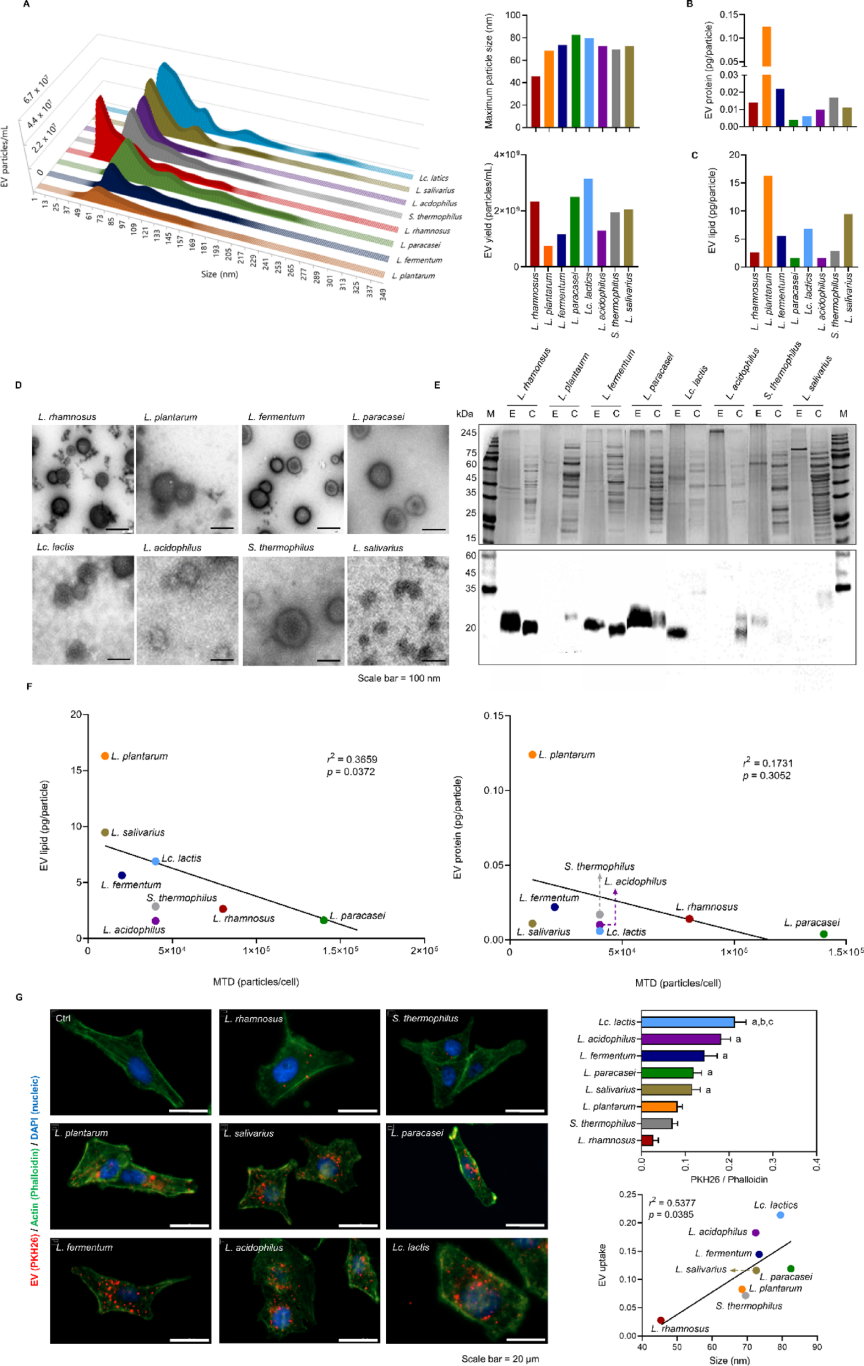
Strain-wide characterization of bEVs. **A**. bEV size distribution and yield. The 3D graph depicts bEV secretion by the bacterial strain. X-axis: bEV diameter (nm), y-axis: bEV strain, z-axis: total bEV yield. **B**. Protein content of each bEV. **C**. Lipid content of each bEV. **D**. bEV morphology by TEM. Scale bar = 100 nm. **E**. Protein profile and LTA expression of each bEV. The upper panel shows Coomassie blue-stained protein profiles of bEVs, and the lower panel presents LTA expression levels. M: molecular weight marker, E: bEV, C: cell lysate. **F**. Correlations of bEV protein and lipid contents with cytotoxicity. X-axis: MTD of bEVs; y-axis: lipid amount (left) and protein amount in bEVs (right). **G**. bEV uptake in KEL FIB cells. Immunofluorescence images of cells show PKH26-labeled bEVs in red, actin in green, and nuclei in blue. Ctrl: vehicle control. Scale bar = 20 μm. The graph results depict bEV uptake efficiency (top) and the correlation between bEV size and uptake (bottom). The data are presented as the means ± SDs; *n* = 11, ^a^*p* < 0.05 vs. *L. rhamnosus*, ^b^*p* < 0.05 vs. *S. thermophilus*, ^c^*p* < 0.05 vs. *L. plantarum*; one-way ANOVA followed by Dunnett’s test.

We characterized bEVs from eight gram-positive bacterial strains of the order *Lactobacillales* in the late exponential growth phase. The bEV size and particle number of each strain analyzed by nanoparticle tracking analysis (NTA) revealed that the bEV from *Lactobacillus paracasei* (*L. paracasei*) was the largest at 82.5 nm, followed by bEV from *Lactococcus lactis* (*Lc. lactis*) at 79.5 nm, and bEV from *Lactobacillus rhamnosus* (*L. rhamnosus*) was the smallest at 45.5 nm. The bEV yield was highest for bEV from *Lc. lactis* at 3.2 × 10^9^ particles/mL, followed by bEV from *L. paracasei* at 2.5 × 10^9^ particles/mL, and lowest for bEV from *L. plantarum* at 7.5 × 10^8^ particles/mL (Fig. 1A). The protein content of bEVs produced by the bacterial strain was highest for bEVs from *L. plantarum* at 0.124 pg/particle, followed by bEVs from *L. fermentum* at 0.022 pg/particle, and lowest for bEVs from *L. paracasei* at 0.004 pg/particle (Fig. 1B). The lipid content of bEVs produced by the bacterial strain was highest for bEVs from *L. plantarum* at a concentration of 16.3 pg/particle, followed by bEVs from *Lactobacillus salivarius* (*L. salivarius*) at 16.3 pg/particle, and lowest for bEVs from *Lactobacillus acidophilus* (*L. acidophilus*) at 1.6 pg/particle (Fig. 1C). From these primary characterizations, no significant correlation was observed between bEV size and yield (*r*^2^ = 0.079, *p* = 0.501). An inverse relationship between bEV yield and protein/lipid cargo contents was observed in some strains but not in most strains (*r*^2^ < 0.343, *p* > 0.114). Transmission electron microscopy (TEM) examination revealed that bEVs were 40 to 110 nm in diameter, which was similar to the NTA results. They appeared as round or cup-shaped vesicles with a distinct lipid bilayer membrane without visible organelles or inclusions (Fig. 1D). The profiling of bEV proteins revealed a simpler pattern, distinct from that of cell lysates, and protein markers were observed that could be representative of each bEV: a 42 kDa protein marker for *L. rhamnosus*, a 75 kDa marker for both *L. plantarum* and *L. fermentum*, a 43 kDa marker for *L. paracasei*, 50 and 75 kDa protein markers for *L. lactis*, a 45 kDa marker for *L. acidophilus*, a 70 kDa marker for *Streptococcus thermophilus* (*S. thermophilus*), and a 100 kDa marker for *L. salivarius* (Fig. 1E, Supplementary Fig. 1A). Lipoteichoic acid (LTA), a membrane-anchored glycolipid unique to gram-positive bacteria, serves as a useful biomarker for bacterial identification and for characterizing bEVs^33^; LTA is approximately 20 kDa in size, and is expressed in most bEVs and cell lysates (Supplementary Fig. 1B). In some bacterial strains, it was preferentially enriched in bEVs or cells, whereas in *L. plantarum*, it was not observed in either. This may be attributed to structural and expression differences in LTA between different bacterial strains and/or limited strain specificity of the applied anti-LTA antibody.

Next, for the application of bEVs, we evaluated the cytotoxicity of bEVs in HEK293T cells, keloid fibroblasts (KEL FIB), and LX-2 cells. Each bEV was applied at increasing concentrations of up to 16 × 10^4^ particles per cell, and its cytotoxicity was evaluated in multiple cell types. In HEK293T cells, bEVs from *L. paracasei* presented the lowest cytotoxicity, with a maximum tolerated dose (MTD) of 14 × 10^4^ particles per cell. This was followed by bEV from *L. rhamnosus*, with an MTD of 8 × 10⁴ particles per cell. The bEVs from *L. plantarum* and *L. salivarius* exhibited the highest cytotoxicity, with an MTD of 1 × 10⁴ particles per cell (Supplementary Fig. 2A). In contrast, in KEL FIB cells, no cytotoxicity was observed even at the highest tested dose of 16 × 10⁴ particles per cell, which was thus determined as the MTD for this cell type (Supplementary Fig. 2B). bEVs from *L. plantarum* and *Lc. lactis* at 16 × 10^4^ particles/cell significantly reduced LX-2 cell viability (*p* < 0.05 and *p* < 0.001, respectively) (Supplementary Fig. 2C). These results indicate that bEVs may exhibit varying cytotoxic effects depending on the cell type, as observed across the three tested cell lines. We evaluated the correlations between the MTDs of each bEV and its protein and lipid contents to determine which of the cargoes of bEV was the determinant of cytotoxicity. Linear regression analysis revealed that the MTD of bEVs in HEK293T cells significantly decreased with increasing lipid content (*r*^2^ = 0.3659, *p* = 0.0372), although there was no correlation with protein content (*r*^2^ = 0.1731, *p* = 0.3052) (Fig. 1F). These results support the previously suggested lipotoxic effect of bEV in part^34,35^. We evaluated the delivery efficiency of each bEV to KEL FIB cells. Uptake varied significantly among strains. *Lc. lactis* bEVs showed the highest uptake, approximately 7.8-fold greater than that of *L. rhamnosus* (*p* < 0.001), followed by *L. acidophilus* at 6.6-fold (*p* < 0.001), *L. fermentum* at 5.3-fold (*p* < 0.05), *L. paracasei* at 4.4-fold (*p* < 0.01), and *L. salivarius* at 4.2-fold (*p* < 0.01). No significant differences were observed for *L. plantarum* or *S. thermophilus* compared with *L. rhamnosus* (Fig. 1G). Uptake levels were moderately positively correlated with bEV size (*r*² = 0.5377, *p* = 0.0385). Therefore, the characterization of bEVs from eight gram-positive bacterial strains revealed significant strain-specific differences in size, yield, protein content, and lipid content, with larger bEVs showing greater delivery efficiency and lipid content being a major factor in cytotoxicity.

### Identification of putative indications of bEVs

**Fig. 2:**
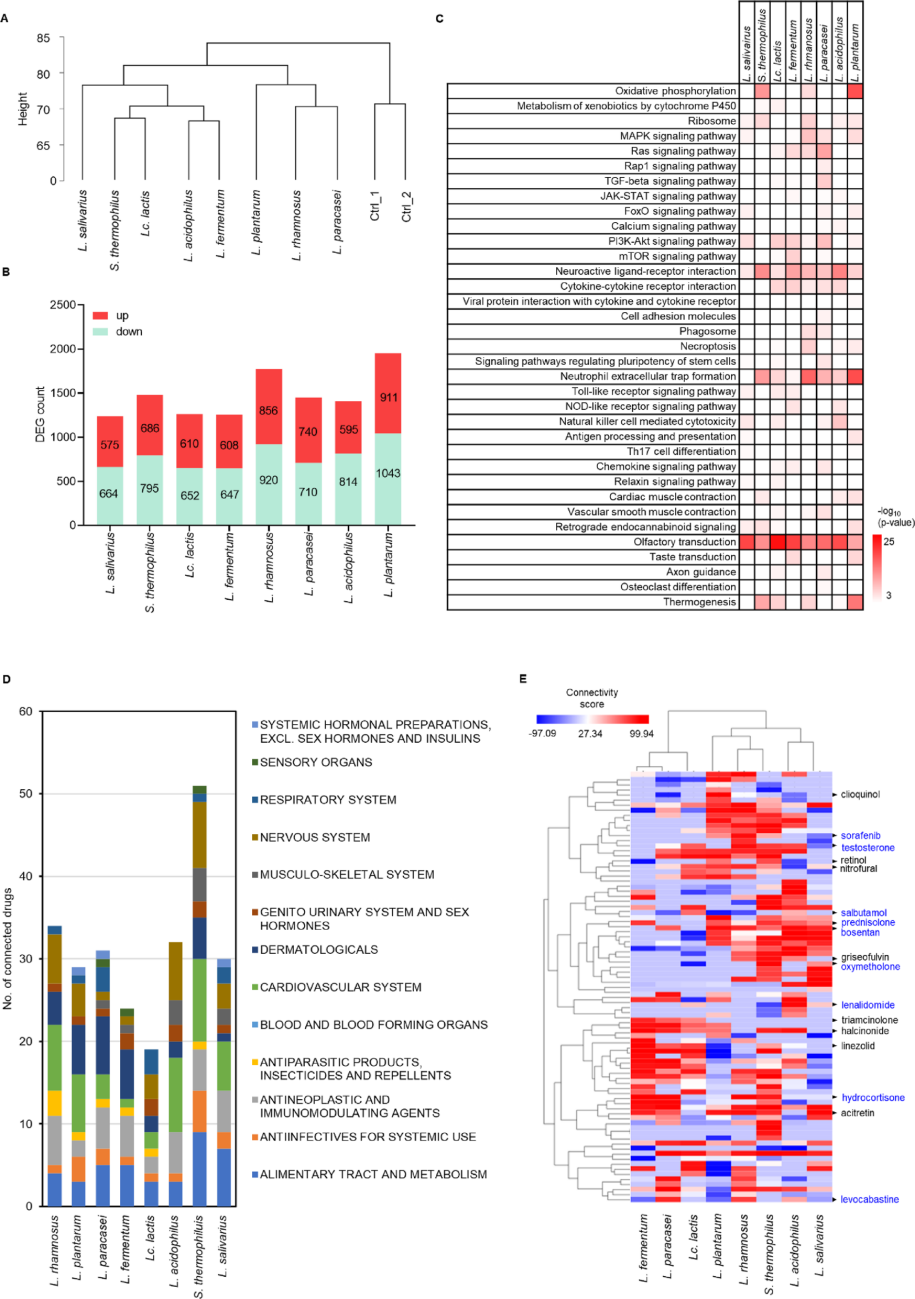
Identification of putative indications for bEVs. **A**. Hierarchical clustering of the bEV-induced transcriptome. The dendrogram presents clustering results inferred from the Euclidean distance between bEV-induced transcriptomes. **B**. bEV-induced DEGs. Each stacked bar represents the number of genes upregulated (red) or downregulated (green) by bEVs. Fold change cutoff = 1.5. **C**. KEGG pathway enrichment analysis. The heatmap depicts the log-transformed *p* values of KEGG pathways enriched with bEV-induced DEGs. **D**. CMap analysis of bEVs. The stacked bar shows the number of drugs classified at ATC level 1 connected to each bEV. The indications of ATC level 1 drugs are presented by color on the right side of the graph. **E**. Hierarchical clustering of bEV-connected drugs. The heatmap represents the connectivity scores between bEVs and drugs, ranging from 99.98 (red) to -97.09 (blue). Drugs in black text indicate skin health therapeutics, and drugs in blue text indicate nonskin health therapeutics.

Pharmacological indications of bEVs were predicted by a drug repositioning strategy using the bEV-induced transcriptomic response. HEK293T cells were treated with each bEV at the MTD and then subjected to global gene expression profiling analysis. Owing to their well-characterized transcriptomic responses and reproducibility in exploratory studies, HEK293T cells were chosen as an initial model for understanding the global effects of bEVs. Hierarchical clustering based on transcriptomic profiles revealed that *L. rhamnosus* and *L. paracasei* clustered together, and were separate from *S. thermophilus* and *Lc. lactis* (Fig. 2A), reflecting their phylogenetic relationships (Supplementary Fig. 3). Differentially expressed gene (DEG) analysis revealed that the number of genes whose expression changed varied by strain, with *L. plantarum* resulting in the greatest number of DEGs, *L. rhamnosus* an intermediate number, and *L. salivarius* the fewest (Fig. 2B). The bEV-induced DEGs were subjected to KEGG pathway enrichment analysis. Neuroactive ligand‒receptor interactions (*p* < 9.4 × 10^−−6^), olfactory transduction pathways (*p* < 1.3 × 10^−−4^), and neutrophil extracellular trap formation (*p* < 2.5 × 10^−−11^) were significantly enriched in most bEVs, regardless of the strain of origin. On the other hand, glycerophospholipid metabolism (*p* = 9.1 × 10^− 7^) in *L. rhamnosus*, cholesterol metabolism (*p* = 1.1 × 10^− 6^) in *S. thermophilus*, and AMPK signaling (*p* = 5.3 × 10^− 6^) in *L. paracasei* were significantly enriched in a strain-specific manner (Fig. 2C, Supplementary Table 1).

To estimate the putative indications of each bEV, bEV-induced DEGs were used as queries for CMap analysis, a transcriptomic database that connects gene expression signatures to potential therapeutic agents. The output of CMap analysis was limited to approved drugs with unique ATC codes for further analysis (Supplementary Table 2). A detailed examination of the connected drugs classified at ATC level 1 for each bEV revealed that the bEV from *S. thermophilus* had the greatest variety and highest number of recognized drugs, followed by the bEVs from *L. rhamnosus*, *L. fermentum*, and *L. salivarius*. In contrast, the bEV from *Lc. lactis* had the smallest number and variety of identified drugs (Fig. 2D). Topical application and injection are two commonly explored routes for nanoparticle-based therapies. On this basis, we categorized the approved drugs from the CMap analysis by route of administration and indication to select the most suitable indications for bEVs. The results revealed that hydrocortisone (bEV from *L. fermentum* with a connectivity score of 99.92) and levocabastine (*Lc. lactis*, 95.95) had high connectivity scores as immunomodulators; sorafenib (*L. rhamnosus*, 96.46), an anti-neoplastic agent; and oxymetholone (*S. thermophilus*, 98.51) and testosterone (*L. rhamnosus*, 96.76), muscle growth agents. High scores were also noted for retinol (*L. fermentum* and *L. acidophilus*, 99.46 and 95.10, respectively), acitretin (*L. rhamnosus*, 96.15), and triamcinolone (*S. thermophilus*, 97.77) for skin health benefits (Fig. 2E). Finally, we attempted to decipher the pharmacological effects of each bEV for its proposed indication on the basis of KEGG pathway enrichment analysis of bEV-induced DEGs. bEVs from *L. rhamnosus* were predicted to activate focal adhesion activity (*p* = 4.6 × 10^4^), Ras (*p* = 4.2 × 10^7^), and PI3K-Akt signaling pathway (*p* = 4.6 × 10^4^), which are associated with skin tightening and elasticity^36,37^. bEVs from *L. fermentum* showed activation of PI3K-AKT (*p* = 9.0 × 10^8^) and mTOR pathways (*p* = 9.2 × 10^8^), along with suppression of the MAPK pathway (*p* = 4.9 × 10^4^), potentially enhancing collagen synthesis and inhibiting melanogenesis^38,39^. bEVs from *L. acidophilus* were predicted to increase JAK-STAT signaling (*p* = 1.8 × 10^4^), whereas those from *S. thermophilus* increased ribosomal activity (*p* = 5.3 × 10^7^) and suppressed oxidative phosphorylation (*p* = 8.0 × 10^13^), which are associated with increased collagen production^40–42^. This integrated analysis allowed us to define the clinical benefit of each bEV and to understand the molecular mechanisms of action that may explain these pharmacologic effects. Therefore, bEVs from *L. rhamnosus*, *L. fermentum*, *L. acidophilus*, and *S. thermophilus* were selected to verify their skin health benefits.

### Enhanced collagen production by bEVs

**Fig. 3:**
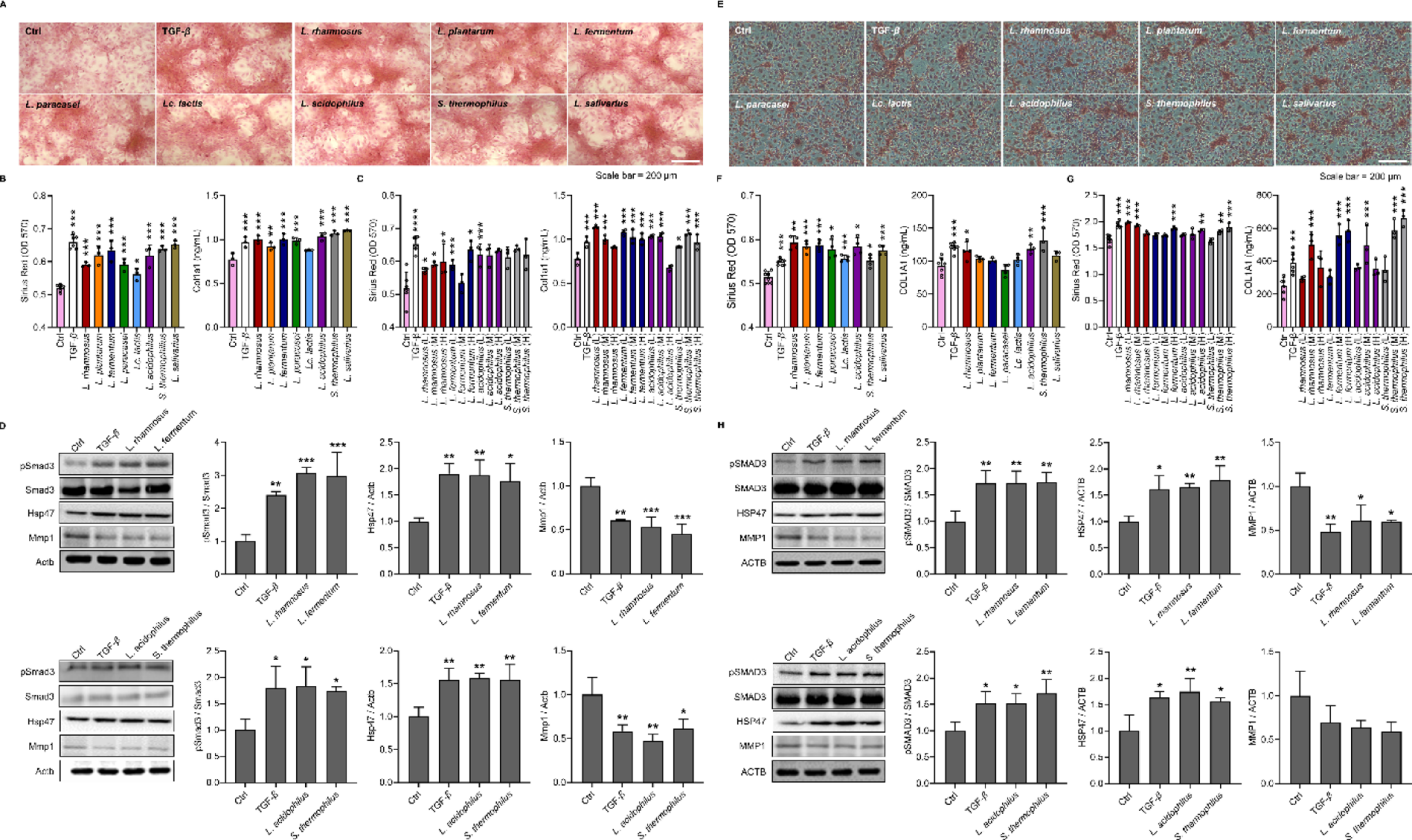
Enhanced collagen production by bEVs. **A**. Collagen staining in bEV-treated NIH3T3 cells. Collagen is stained with Sirius Red. Scale bar = 200 μm. **B**. Collagen quantification in NIH3T3 cells. **C**. Dose-dependent collagen production in NIH3T3 cells by selected bEVs. The extracted and quantified Sirius Red is shown on the left, and the amount of collagen is measured by ELISA on the right. **D**. Collagen synthesis and key molecules involved in collagen homeostasis in NIH3T3 cells. TGF-β is used as a positive control. **E**: Collagen staining in bEV-treated LX-2 cells. Scale bar = 200 μm. **F**: Collagen quantification in LX-2 cells. **G**. Dose-dependent collagen production in LX-2 cells by selected bEVs. **H**. Collagen synthesis and homeostasis axis activity in LX-2 cells. H, M, and L indicate high, middle, and low concentrations of bEVs, respectively, corresponding to three times, one time, and one-third of MTD. The data are presented as the means ± SDs; *n* = 3 ~ 8; *p < 0.05. **p < 0.01, ***p < 0.001 vs. vehicle control (Ctrl), one-way ANOVA followed by Dunnett’s test.

Cell-based collagen production experiments were conducted to prove the skin health benefits of bEVs predicted by in silico analysis. NIH3T3 cells were treated with bEVs at MTD, and the amount of collagen produced was quantified via Sirius Red staining (Fig. 3A). The results showed that TGF-β treatment increased collagen production by 1.27-fold compared with vehicle (*p* < 0.001). bEVs from *L. rhamnosus*, *L. plantarum*, *L. fermentum*, *L. paracasei*, *Lc. lactis*, *L. acidophilus*, *S. thermophilus*, and *L. salivarius* also significantly increased collagen production (*p* < 0.05) (Fig. 3B). ELISAs confirmed that collagen levels were elevated with bEV treatment compared with vehicle control, although the absolute amount of collagen varied across strains. bEVs from *L. rhamnosus*, *L. fermentum*, *L. acidophilus*, and *S. thermophilus*, which showed consistent increases across assays, were selected for further evaluation. We evaluated the dose-dependent effects of the selected bEVs on collagen production at one-third of the MTD (low concentration), the MTD (middle concentration), and three times the MTD (high concentration). Sirius Red staining revealed that some bEVs increased collagen production at specific concentrations, but the trend was not consistently dose-dependent (Fig. 3C). For example, *L. rhamnosus* increased collagen production by 1.14-fold at the middle and 1.15-fold at high concentrations (*p* < 0.05), whereas *L. fermentum* and *S. thermophilus* increased collagen production mainly at low or middle concentrations. In addition, the ELISA results showed an inconsistent pattern across doses. *L. rhamnosus* had the greatest effect on collagen levels at low concentration, and *L. acidophilus* had the smallest effect at high concentration. Taken together, while individual treatments occasionally resulted in statistically significant increases, a consistent dose-response relationship was not observed. We then examined the expression of key regulators of collagen homeostasis, including Smad3, Hsp47, and MMP1, in samples treated with high concentrations of bEVs, to elucidate the underlying mechanism of collagen synthesis^40^. The results showed that bEVs from *L. rhamnosus* significantly increased pSmad3 and Hsp47 levels while reducing Mmp1 levels. Similarly, *L. fermentum* increased pSmad3 and Hsp47 and decreased Mmp1. bEVs from *L. acidophilus* and *S. thermophilus* also modulated these markers in the same direction, albeit to a lesser extent (Fig. 3D, Supplementary Fig. 4). These findings suggest that bEVs promote collagen synthesis by modulating key regulators of collagen production and extracellular matrix homeostasis, and that these effects are comparable to those of TGF-β.

bEV-induced effects on collagen synthesis were also observed in LX-2 cells. Sirius red staining revealed that, compared with the vehicle control, bEVs from *L. rhamnosus* increased collagen production by 1.15-fold (*p* < 0.001), followed by those from *L. fermentum* (1.14-fold, *p* < 0.001), *L. acidophilus* (1.14-fold, *p* < 0.01), and *S. thermophilus* (1.07-fold, *p* < 0.05) (Fig. 3E). ELISA confirmed that bEVs from each bacterial strain increased collagen production: *L. rhamnosus* by 1.25-fold (*p* < 0.005), *L. acidophilus* by 1.28-fold (*p* < 0.01), and *S. thermophilus* by 1.42-fold (*p* < 0.001) (Fig. 3F). Sirius Red staining revealed that several bEVs, including those from *L. rhamnosus*, *L. fermentum*, *L. acidophilus*, and *S. thermophilus*, significantly increased collagen production compared with vehicle control at various concentrations. However, the changes did not follow a consistent dose-dependent trend across the low, middle, and high concentrations. Similarly, the ELISA results confirmed significant increases in collagen levels for all the tested bEVs (*p* < 0.001), with the highest fold-changes observed in the cells treated with bEVs from *L. fermentum* and *S. thermophilus*. However, as with the Sirius Red data, a clear dose-response relationship was not consistently observed (Fig. 3G). Western blotting revealed that pSMAD3 was significantly increased by bEVs from *L. rhamnosus* (1.72-fold, *p* < 0.01), *L. fermentum* (1.74-fold, *p* < 0.01), *L. acidophilus* (1.52-fold, *p* < 0.05), and *S. thermophilus* (1.7-fold, *p* < 0.01), whereas HSP47 was significantly increased by bEV from *L. rhamnosus* (1.6-fold, *p* < 0.01), *L. fermentum* (1.79-fold, *p* < 0.01), *L. acidophilus* (1.74-fold, *p* < 0.01), and *S. thermophilus* (1.56-fold, *p* < 0.05). MMP1 was decreased by bEV from *L. rhamnosus* (0.63-fold, *p* < 0.05), *L. fermentum* (0.60-fold, *p* < 0.05), *L. acidophilus* (0.64-fold), and *S. thermophilus* (0.59-fold) (Fig. 3H, Supplementary Fig. 4). Therefore, these results demonstrated the potential efficacy of bEV as an agent for enhancing collagen synthesis in both human and murine cells. These findings validate the transcriptome-based discovery strategy for bEVs and identify bEVs from *L. rhamnosus*, *L. fermentum*, *L. acidophilus*, and *S. thermophilus* as promising ingredients for skin health.

### bEV and host interactions in collagen production

**Fig. 4:**
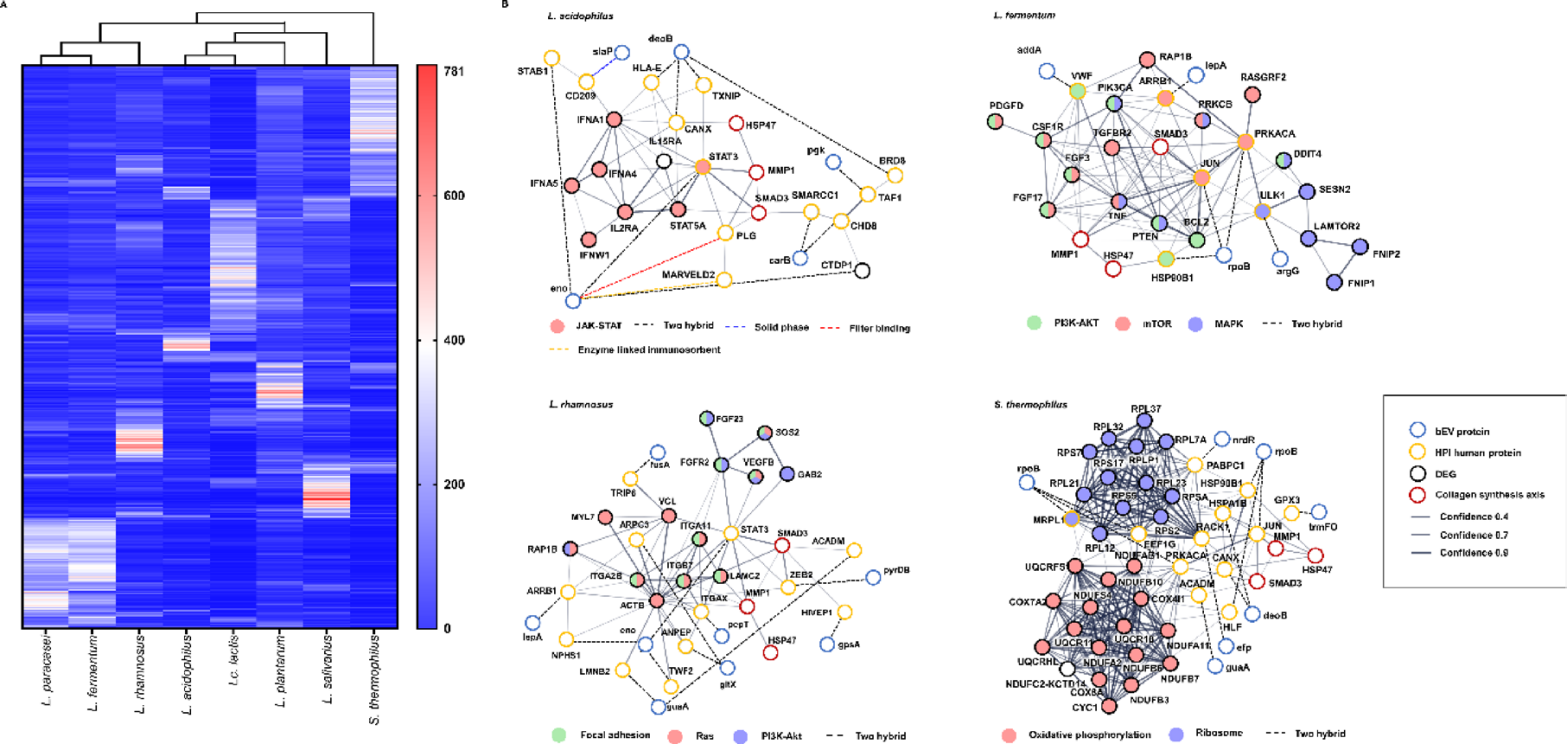
Molecular mechanisms of bEV proteins in collagen synthesis. **A**. Hierarchical clustering of bEV proteins. The dendrogram presents clustering results inferred from the Euclidean distance between bEV proteins. The heatmap represents the abundances of total bEV proteins from 0 (blue) to 781 (red). **B**. Interacting networks of bEV proteins with their human counterparts for collagen synthesis. bEV proteins are depicted as blue empty circles, human proteins interacting with bEV proteins are depicted as yellow empty circles, collagenogenic pathway-enriched bEV-induced DEGs are depicted as black empty circles, and proteins in the collagen synthesis axis are depicted as red empty circles. Each filled circle represents a collagenogenic pathway regulated by bEVs, and the dashed lines represent the experimental methods used to determine the interaction of bEV proteins with human proteins. The solid lines indicate the confidence of protein interactions on the basis of their thickness.

Proteomic analysis was conducted to identify the putative active ingredients in bEVs that promote collagen synthesis. LC‒MS/MS analysis revealed a total of 950 to 967 proteins across all bEV strains, meeting the criteria of high protein false discovery rate (FDR) confidence and at least two unique peptides (Supplementary Table 3). These values represent the total number of proteins identified per strain, with variability depending on the bacterial source. Specifically, 191 strain-specifically abundant proteins were identified in bEVs from *L. paracasei*, 183 from *L. fermentum*, 106 from *L. rhamnosus*, 55 from *L. acidophilus*, 234 from *Lc. lactis*, 116 from *L. plantarum*, 109 from *L. salivarius*, and 222 from *S. thermophilus*. These proteins constitute distinct sets for each bEV, providing a foundation for further analysis of their strain-specific roles and functions (Fig. 4A). For a comprehensive analysis of molecular interactions, we utilized several datasets. We first identified bEV proteins through proteomics analysis and mapped their human interaction partners using the Host-Pathogen Interaction Database (HPIDB), which provides experimentally validated bacterial-human protein-protein interactions. Additionally, we incorporated signaling molecules associated with collagen production, identified through KEGG pathway analysis, along with key molecules involved in collagen synthesis and homeostasis, such as SMAD3, HSP47, and MMP1 ^40^. These integrated datasets were subjected to STRING analysis to elucidate the molecular interaction networks. This analysis suggested that the proteins deoB, slap, carB, ppk, and eno in bEVs from *L. acidophilus* may interact with STAB1, MARVELD2, CD209, HLA-E, CANX, TXNIP, PLG, SMARCC1, CHD8, TAF1, BRD8, and STAT3, potentially influencing the JAK-STAT signaling pathway, which is associated with the regulation of SMAD3, HSP47, and MMP1 ^40,43^. In *L. fermentum*, bEV proteins addA, rpoB, and argG are predicted to interact with VWF, ARRB1, PRKACA, JUN, ULK1, and HSP90B1, potentially modulating the PI3K-AKT and mTOR signaling pathways while downregulating the MAPK pathway^38,44,45^. Several bEV proteins of *L. rhamnosus*, including fusA, lepA, eno, guaA, pepT, gltX, gpsA, and pyrDB, were predicted to interact with human proteins such as TRIP6, ARPC3, STAT3, ACADM, ZEB2, HIVEP1, ITGAX, ANPEP, TWF2, LMNB2, NPHS1, and ARRB1. These interactions suggest possible roles in the upregulation of the focal adhesion, Ras, and PI3K-AKT pathways, and affecting SMAD3, HSP47, and MMP1 activities. In *S. thermophilus*, the bEV proteins such as rpoB, nrdR, and trmFO were predicted to interact with a range of human proteins, including PABPC1, HSP90B1, GPX3, HSPA1B, JUN, RACK1, EEF1G, MRPL1, PRKACA, CANX, ACADM, and HLF. These interactions may influence oxidative phosphorylation and ribosomal activity, and potentially regulate SMAD3, HSP47, and MMP1 (Fig. 4B). These findings suggest that bEV proteins may influence collagen synthesis through specific signaling pathways and molecular interactions, as indicated by integrated bioinformatic analyses.

## Discussion

The characterization of bEVs from eight *Lactobacillales* strains in this study revealed significant variability in bEV size, yield, protein content, and lipid content. This variability highlights the diverse biogenesis and secretion mechanisms of bEVs across strains, contributing to the observed differences. While our findings on size variability and protein composition patterns align with the literature on bEVs^46,47^our study uniquely reveals strain-specific characteristics that have not been extensively described or documented in previous studies. The identification of strain-specific protein markers and lipid profiles suggests potential roles for bEVs in intercellular communication and host-pathogen interactions, with larger bEVs being more efficient in delivery and lipid content significantly contributing to cytotoxicity. We also observed that the LTA distribution varies significantly between bacterial strains and their EVs. Some strains, such as *L. paracasei*, present high LTA levels in both cells and bEVs, whereas others, such as *L. plantarum*, present high LTA levels only in cells. Conversely, *Lc. lactis* and *S. thermophilus* present LTA exclusively in bEVs. While LTA is a common feature of gram-positive bacteria, its expression and structure can vary greatly among different strains, with some strains even lacking LTA or having altered LTA expression^48,49^. Notably, this study revealed that higher LTA expression levels in bacterial cells tended to be associated with increased protein and lipid contents in the corresponding bEVs, although the statistical significance of this correlation was marginal. However, LTA expression appeared to have little influence on other bEV characteristics such as size or yield. Overall, our findings highlight significant strain-specific variability in bEV composition among gram-positive bacteria, suggesting diverse biogenesis mechanisms and potential functional differences in intercellular communication and host-pathogen interactions.

EVs, including bEVs, possess significant potential as biomarkers and therapeutic agents because of their ability to carry diverse molecular cargo. However, the identification of clinical indications for bEVs is challenging, and traditional approaches such as preclinical studies and target disease selection can be limiting in advancing these innovative approaches. CMap offers a valuable opportunity to uncover new indications for bEVs. While CMap is typically utilized for drug repurposing^50,51^it has also been shown to be effective in identifying new therapeutic applications. Although the use of HEK293T-based transcriptomes may raise concerns regarding generalization, many transcriptomic responses reflect core biological pathways conserved across cell types. For example, studies have used CMap to identify potential therapeutic uses for 3-dimensional cultured mesenchymal stem cell-derived EVs^52^explore potential applications of telmisartan in nonalcoholic steatohepatitis (NASH)^53^and discover new clinical uses for this new chemical entity in treating neutrophilic bronchial asthma^54^. We applied CMap to identify putative indications for bEVs via their transcriptomic signatures. CMap linked these bEVs to skin care treatments, specifically collagen production, and identified highly relevant drugs such as retinol, acitretin, and triamcinolone. The collagenogenic potential of bEVs predicted by CMap analysis was further proven experimentally. Specifically, bEVs derived from *L. rhamnosus*, *L. fermentum*, *L. acidophilus*, and *S. thermophilus* enhanced collagen production in human and mouse collagen-producing cells at specific concentrations, although a consistent dose-dependent pattern was not observed. Additionally, these bEVs regulate the expression of key proteins involved in collagen synthesis, including SMAD3, HSP47, and MMP1, thereby promoting collagen production. To our knowledge, this is the first study to use CMap to discover indications for bEVs and validate them. This study provides a new alternative to traditional bEV research and is expected to be a valid way to accelerate the clinical utilization of bEVs.

One significant challenge in elucidating the mode of action of bacteria and their products is the lack of suitable methods to understand cross-species interactions and identify active bacterial ingredients. The HPIDB addresses this issue by offering a comprehensive repository of experimentally validated human protein‒bacterial protein interactions. Its ability to interface with other databases and analytical tools, such as STRING, further increases our understanding of the molecular interactions at play, facilitating both basic and applied research in elucidating bacterial mechanisms of action. In this study, we utilized these tools to investigate how proteins from the bEVs of *L. acidophilus* interact with human proteins, influencing the regulation of the JAK-STAT pathway. Key interactions include slaP with CD209 and deoB with HLA-E, both of which are involved in JAK-STAT activation^55^. The pathway is also regulated by the deoB-TXNIP interaction^56^. Additionally, carB interacts with SMARCC1, a component of the SWI/SNF chromatin remodeling complex, whose loss increases chromatin accessibility at JAK-STAT target loci^57^. Reducing TAF1 decreases JAK-STAT activity, indicating its role as a positive regulator of this pathway^58^. STAT3, a crucial transcription factor, is activated by the JAK-STAT pathway^59^. This pathway also involves proteins involved in ECM remodeling and fibrosis, such as SMAD3, HSP47, and MMP1, thereby promoting collagen synthesis^40,43^. Our investigation into bEV proteins from *L. fermentum* also revealed interactions with human proteins that regulate the PI3K-AKT and mTOR signaling pathways, enhancing collagen synthesis. Key interactions include addA with VWF, which regulates the PI3K-AKT and MAPK pathways^60,61^; rpoB with HSP90B1 and PRKACA, where HSP90B1 regulates PI3K-AKT-mTOR signaling^62^; PRKACA acts as a negative regulator of mTORC1 ^63^; and argG with ULK1, which is a negative regulator of mTORC1^64^. The PI3K-AKT pathway enhances SMAD3 transcriptional activity, increasing collagen I expression in human cells^36^and mediates TGF-β2-induced type I collagen synthesis^37^. AKT acts as a negative regulator of MMP1 gene expression in dermal fibroblasts^65^whereas mTOR inhibition increases MMP1 expression^38^. The interaction between SMAD3 and mTORC1 promotes HIF-1 expression, which is crucial for TGF-β-induced collagen expression^39^. The AKT/mTOR pathway also regulates collagen synthesis in tendon cells in response to mechanical loading^66^. Similarly, in *L. rhamnosus*, bEV proteins interact with human proteins, leading to the upregulation of the focal adhesion, Ras, and PI3K-AKT pathways, which increase collagenogenic activity by influencing key regulators of collagen synthesis and remodeling^44,45^. In *S. thermophilus*, bEV proteins regulate oxidative phosphorylation and ribosomal activity through their interactions with human proteins, thereby modulating these collagen synthesis regulators and promoting collagenogenic activity^41,42^. Thus, this study offers valuable insights into addressing the challenge of identifying interspecies interactions by elucidating the mechanisms of action of bEV proteins with human proteins via an integrated bioinformatics approach. By revealing how bacterial products induce collagen production through key signaling pathways in the host, we provide potential therapeutic targets to enhance collagen synthesis and improve skin health.

While this study is valuable for its innovative approaches and new findings that have not been previously reported, it also has several limitations. First, although tangential flow filtration (TFF) accompanied with buffer exchange was employed for bEV isolation, this method may not have been fully sufficient to remove all soluble bacterial products. This limitation means that the high protein and lipid contents observed in some strains may partially reflect residual soluble components rather than true bEV cargo. This technical limitation should be taken into account when interpreting the strain-specific characteristics and functional effects of bEVs reported in this study. Second, the discovery of active bacterial ingredients beneficial to collagen production through integrated bioinformatic analyses is highly important; however, no functional evaluation of the discovered ingredients or interactions has been performed. Future research should focus on validating these bioinformatic predictions through experimental studies to confirm the efficacy of the identified components in promoting collagen synthesis by bEVs. Third, expanding the range of bacterial strains and exploring different growth phases could further increase our understanding of bEV production and characteristics. By including a more diverse selection of bacterial strains, we can capture a broader representation of the diversity and variability inherent in bEVs. This comprehensive approach could help identify strain-specific features and potentially reveal novel bEVs with unique properties. Additionally, studying different growth phases would provide insights into how bEV production and composition change over time, offering a more dynamic understanding of bEV biogenesis. Lastly, incorporating a more comprehensive cargo analysis, including RNA and metabolite analyses, could uncover additional layers of complexity and potential functionality within bEVs. This holistic approach to cargo analysis provides a deeper understanding of the molecular composition of bEV and its implications for bEV activity. By exploring these additional components, future studies can better elucidate the full range of biological effects mediated by bEVs, paving the way for more targeted and effective therapeutic applications.

In conclusion, we characterized bEVs from eight gram-positive bacterial strains, revealing significant strain-specific differences. Bioinformatic analysis was used to predict the potential of bEVs for enhancing collagen production, and the results were experimentally validated. Additionally, we elucidated the interaction mechanisms between bEV proteins and the host, suggesting a molecular basis for their potential skin health benefits. These findings highlight the therapeutic potential of bEVs, present a successful approach for discovering new indications for bEVs and provide a foundation for novel skin care applications.

## Methods

### Bacterial cell culture

Eight *Lactobacillales* strains, including *L. paracasei*, *L. acidophilus*, *Lc. lactis*, *L. fermentum*, *L. rhamnosus*, *L. salivarius*, *S. thermophilus*, and *L. plantarum*, were isolated from diverse sources such as fermented dairy products and plants, and their strain rights and cultivation were managed by HK inno.N (Korea). These gram-positive bacterial strains were selected based on their widespread use in the probiotics and food industries, established safety profiles, and phylogenetic diversity across representative genera. Seedlings were grown in 40 mL of MRS medium (Becton Dickinson, cat#288110) in TubeSpin Bioreactors (TPP Techno Plastic Products AG, cat#87050) after inoculation and incubated in an anaerobic chamber at 37 ± 1 °C for 15 h. The seedling cultures were transferred into 900 mL of MRS medium in a 1 L storage bottle (Corning, cat# CLS430518) to reach an absorbance of 0.04 at 600 nm (OD600) and incubated in an anaerobic chamber at 37 ± 1 °C, reaching the strain-specific late phase of exponential growth was reached within 12 h. At the end of incubation, the medium was centrifuged at 4,000 rpm for 15 min to recover 600 mL of the supernatant and then filtered through a 0.2 μm pore size membrane filter (Corning, cat#CLS431174). The sterilized culture supernatant was frozen at -20 °C until use.

### Mammalian cell culture

HEK293T (ATCC, cat#CRL-3216) and LX-2 (Merck Millipore, cat#SCC064) cells were cultured in DMEM (Gibco, cat#11965092) supplemented with 10% (v/v) FBS (Gibco, cat#16000044) and 1% (v/v) antibiotic-antimycotic solution (Gibco, cat#15240062). KEL FIB (ATCC, cat#CRL-1762) and NIH3T3 (ATCC, cat#CRL-1658) cells were cultured in DMEM supplemented with 10% (v/v) FBS, 1% (v/v) antibiotic-antimycotic solution, and 2 mM GlutaMAX (Gibco, cat#35050061). All the cell cultures were maintained at 37 °C in a humidified atmosphere containing 5% CO₂.

### TFF

The 600 mL culture supernatant was thawed, and bEVs were harvested via TFF. This was performed via a Sartoflow Smart TFF system (Sartorius) equipped with a Sartocon Slice 200 membrane with a molecular weight cutoff of 100 kDa (Sartorius, cat#3081466802E-SW). During the process, the transmembrane pressure was maintained at 0.5 bar, the feed pressure was set to 1 bar, and the retentate pressure was maintained at 0 bar. The supernatant was concentrated 20-fold to reduce the volume and enrich for bEVs. Buffer exchange was then performed using more than four volumes of PBS (Thermo Fisher Scientific, cat# 10010023) relative to the bEV concentrate to effectively remove residual soluble contaminants. Following the diafiltration process, the bEV concentrate was purified by passing it through a 0.2 μm filter (Sartorius, cat#17823) to remove any remaining large particles or contaminants.

### NTA

bEV concentrate was dissolved in PBS at dilution ratios ranging from 1:20 to 1:100. The diluted EV samples were analyzed via a NanoSight NS300 instrument (Malvern Panalytical). The instrument settings were configured with a capture screen gain of 2 and a detection threshold screen gain of 10. Each sample was captured three times, and the results were averaged for the size distribution and concentration of bEVs.

### Quantification of bEV protein

For protein extraction, the bEV concentrate was mixed at a 2:1 ratio with RIPA buffer (Thermo Fisher Scientific, cat# 89900) containing 1% (v/v) protease inhibitor, 1% (v/v) phosphatase inhibitor, and 1% (v/v) EDTA (GenDEPOTcat# P3100-001). The mixture was thoroughly vortexed and incubated at 4 °C for 20 min with intermittent shaking at 140 rpm. Following incubation, the mixture was centrifuged at 15,493 × g for 15 min at 4 °C, and the supernatant containing the extracted proteins was collected. The protein concentration was quantified via a Pierce BCA protein assay kit (Thermo Fisher Scientific, cat# A55864). The absorbance was measured at 570 nm via a Cytation 5 plate reader (BioTek), with three replicate measurements.

### Quantification of bEV lipids

The bEV was sonicated via a Bioruptor Plus sonication device (Diagenode) at high power for 8 cycles of 15 s of sonication followed by a 15 s interval at 4 °C. For the phosphoric acid-vanillin assay for bEV lipid quantification, 0.120 g of vanillin (Sigma‒Aldrich, cat#V1104) was dissolved in 20 mL of distilled water, followed by the addition of 80 mL of 85% (v/v) ortho-phosphoric acid (Sigma‒Aldrich, cat#695017). Triolein standards (Sigma‒Aldrich, cat# Y0001113) were prepared in chloroform at concentrations of 0, 1, 2, 3, and 4 mg/mL. For each standard and the bEV samples, 100 µL of solution was added to separate 15 mL tubes (SPL Life Sciences, cat#50015), the chloroform was evaporated via nitrogen flux (Goojung), and 2 mL of 18 M sulfuric acid (Sigma‒Aldrich, cat#258105) was added. The tubes were incubated in a 95 °C water bath for 10 min and cooled for 5 min at room temperature, followed by the addition of 5 mL of the prepared phosphoric acid-vanillin mixture and further incubation at 37 °C for 15 min. The optical density was measured at 530 nm via a glass cuvette against a reference tube containing 100 µL of water. The absorbance values of the triolein standards were plotted to create a standard curve, which was then used to determine the lipid concentration in the bEV samples on the basis of their absorbance values.

### TEM

For electron microscopy analysis of bEVs, 5 µL of bEV concentrate was loaded for 1 min on a 300-mesh carbon-coated grid (Sigma‒Aldrich, cat# 931519). The grid was gently blotted with filter paper, and bEVs were negatively stained with 2% (v/v) uranyl acetate (EMS, cat#22400) for 5 s. The uranyl acetate on the grid was blotted off, and the grid was left to dry. The bEV-loaded grid was analyzed under a JEM1010 transmission electron microscope (JEOL) operating at 80 kV.

### bEV uptake

KEL FIB cells were cultured on chamber slides (Thermo Fisher Scientific, cat# 177372PK) at a seeding density of 1 × 10^4^ cells per slide. The bEVs were labeled with the PKH26 Red Fluorescent Cell Linker Kit (Sigma‒Aldrich, cat# PKH26GL) following the manufacturer’s instructions. The cells were treated with PKH26-labeled bEVs at 1.6 × 10^5^ particles/cell for 6 h and then fixed with 10% formalin (BYLABS, cat# F0196RD) for 10 min at room temperature. The cells were then permeabilized with 0.1% (v/v) Triton X-100 (Sigma‒Aldrich, cat#T8787) in PBS for 5 min. For cytoskeletal staining, the cells were incubated with Alexa Fluor 488 phalloidin (Thermo Fisher Scientific, cat#A12379) for 15 min and counterstained with DAPI (Maravai Life Sciences, cat# H-1200) for 5 min to stain the nuclei. The slides were mounted with ProLong Gold Antifade Mountant (Thermo Fisher Scientific, cat# P36930), and the uptake of bEVs was quantified by analyzing red fluorescence in individual cells via Cytation 5 (BioTek). The fluorescence intensity was measured and analyzed via Gen5 software (BioTek) to determine the extent of bEV uptake by the KEL FIB cells.

### Coomassie blue staining

The bEV protein was loaded onto a 10% (w/v) SDS‒PAGE gel and electrophoresed at 100 V for 90 min via a PowerPac Basic Power Supply (Bio-Rad). The gel was washed twice with distilled water and stained for 20 min with InstantBlue Coomassie Protein Stain (Abcam, cat#ab119211). After washing, the stained gel was imaged via a ChemiDoc MP Imaging System (Bio-Rad) for visualization of the protein bands present in the bEVs.

### Cell viability assay

Cell viability was evaluated via the CyQUANT MTT Cell Viability Assay (Invitrogen, cat# V13154) following the manufacturer’s instructions. Various concentrations of bEVs were applied to HEK293T, KEL FIB, and LX-2 cells for 6 h. After the cells were washed with DPBS, a mixture of 10 µL of MTT and 100 µL of phenol red-free culture medium was added. Following a 4-hr incubation at 37 °C, 100 µL of 0.01 M HCl was added, and the absorbance of each sample was measured at 570 nm via a Cytation 5 instrument (BioTek). The MTD was defined as the highest concentration of bEVs that maintained ≥ 80% cell viability relative to vehicle control.

### Microarray analysis

For transcriptome analysis, HEK293T cells were treated with bEVs at the MTD for 6 h. Total RNA was extracted via the RNeasy Mini Kit (Qiagen, cat# 74104). Triplicate RNA samples were pooled into one composite sample. RNA samples with a RNA integrity number (RIN) ≥ 6.6 were used for cDNA synthesis with the GeneChip WT cDNA Synthesis and Amplification Kit (Applied Biosystems). The synthesized cDNA was fragmented and labeled with biotin via the GeneChip WT Terminal Labeling Kit (Applied Biosystems). Approximately 5.5 µg of labeled cDNA was hybridized to the Affymetrix GeneChip Human Gene 2.0 ST Array (Affymetrix) at 45 °C for 16 h. After hybridization, the arrays were scanned via a GCS3000 scanner (Affymetrix), and data analysis was performed via GeneChip Command Console Software (Affymetrix).

### Proteome analysis

bEVs were precipitated via cold acetone and dissolved in solubilization buffer containing 5% (w/v) SDS and 50 mM TEAB (pH 7.55). A total of 100 µg of protein from each bEV was digested via S-Trap mini spin columns (Protifi) according to the manufacturer’s instructions. The digested peptides were labeled with TMT10plex isobaric label reagents (Thermo Fisher Scientific) and separated via a reversed-phase fractionation liquid chromatography system (Agilent Technologies). Fractionated peptide samples resuspended in 0.1% (v/v) aqueous formic acid solution were subjected to a Q Exactive HF-X hybrid quadrupole-orbitrap mass spectrometer (Thermo Fisher Scientific) coupled with an Ultimate 3000 RSLCnano system (Thermo Fisher Scientific). The peptides were loaded onto a trap column (75 μm × 2 cm) packed with Acclaim PepMap100 C18 resin, separated on an analytical column (EASY-Spray column, 75 μm × 50 cm, Thermo Fisher Scientific), and sprayed onto a nano-ESI source. A top 10 data-dependent method was used to operate the Q Exactive HF-X mass analyzer. Full MS scans were acquired over the m/z range between 400 and 2000 with a mass resolution of 60,000 at m/z 200 and an AGC target value of 5×e6. In the higher-energy collisional dissociation (HCD) collision cell with a normalized collision energy of 32, the ten most intense peaks with charge states ≥ 2 were fragmented, and tandem mass spectra were acquired with a mass resolution of 45,000 at m/z 200 in the Orbitrap mass analyzer. All the raw LC‒MS/MS data were analyzed via Proteome Discoverer 3.0 software (Thermo Fisher Scientific) for protein identification and reporter ion-based quantitation. CHIMERYS was used for the database search (https://www.uniprot.org/), with at least two unique peptides and high protein confidence at a false discovery rate (FDR) of 1%.

### Bioinformatic analysis

To investigate the molecular mechanisms underlying bEV effects, DEGs were identified in HEK293T cells via a fold change cutoff of 1.5. The DEGs were subjected to KEGG pathway enrichment analysis (release 108) (https://www.kegg.jp)^67,68^ to identify significantly altered signaling pathways at *p* < 0.001. Potential indications of bEV were explored via the CMap database (https://clue.io)^69^which contains information concerning transcriptional responses to various pharmacological or genetic interventions in cell lines. The top 30 upregulated and top 30 downregulated DEGs induced by bEVs were used as queries in CMap, applying filters to select approved drugs with an anatomical therapeutic chemical (ATC) code and a connectivity score cutoff of 90. Human proteins that interact with bEV proteins were identified using the HPIDB (version 3.0) (http://www.hpidb.igbb.msstate.edu)^70^. This analysis involves searching for known human proteins that interact with the identified bEV proteins via experimental interaction data from HPIDB. To further understand the biological networks of proteins involved in the affected pathways, the STRING protein‒protein interaction network (version 12.0) (https://string-db.org) was utilized (interaction score ≥ 0.4, FDR *q* value ≥ 0.05). This comprehensive analysis included bEV-induced DEGs; bEV proteins identified from proteomic analysis; human proteins interacting with bEV proteins from HPIDB; and key proteins involved in collagen synthesis and homeostasis, including SMAD3, HSP47, and MMP1.

### Sirius red staining

LX-2 and NIH3T3 cells were seeded at a density of 1 × 10^5^ cells per well in poly-D-lysine-coated 24-well plates (SPL Life Sciences, cat# 30024) with 500 µL of Opti-MEM (Thermo Fisher Scientific, cat# 31985062). The cells were treated with either 10 ng/mL TGF-β (Sigma‒Aldrich) or bEVs at various concentrations and then incubated for 24 h. The cells were subsequently washed with DPBS and fixed with Bouin’s solution (Sigma‒Aldrich) for 1 h at room temperature. Picrosirius red solution (Abcam, cat# ab246832) was used to stain the cells for 2 h at room temperature. The cells were then washed with 0.01 N HCl (Sigma‒Aldrich, cat#320331) to remove the unbound dye, and the bound dye was eluted with 0.1 N NaOH (WELGENE, cat#ML022-01). The absorbance was measured at 570 nm via a Cytation 5 plate reader (BioTek).

### Western blotting and LTA measurement

Proteins were separated on a 10% (w/v) SDS-PAGE gel and transferred to a 0.45 μm PVDF membrane (Thermo Fisher Scientific, cat# 88518). The membrane was blocked with 5% (w/v) BSA (Sigma‒Aldrich, cat# 9048-46-8) in Tris-buffered saline with 0.1% (v/v) Tween-20 (TBS-T) for 1 h at room temperature. The membrane was then incubated overnight at 4 °C with the following primary antibodies: LTA (1:250, Abcam, cat#ab267414), SMAD3 (1:1000, Abcam, cat#ab40854), pSMAD3 (1:1000, Abcam, cat#ab52903), MMP1 (1:500, Abcam, cat#ab134184), HSP47 (1:1000, Santa Cruz Biotechnology, cat#sc-5293), and ACTB (1:1000, Santa Cruz Biotechnology, cat#sc-47778). After primary antibody incubation, the membrane was washed with TBS-T and then incubated for 1 h at room temperature with the following secondary HRP-conjugated antibodies: goat anti-mouse IgG (1:5000, Invitrogen, cat#31430) and goat anti-rabbit IgG (1:5000, Invitrogen, cat#31460). Signal detection was performed via a chemiluminescence kit (Bio-Rad), and the proteins were analyzed with ImageJ (https://imagej.nih.gov/ij).

### ELISA

To assess the concentration of collagen type I released from NIH3T3 and LX-2 cells following bEV treatment, the medium supernatant was harvested and analyzed. The concentration of procollagen type I C-peptide (PIP) in the supernatant was measured via the PIP Enzyme Immunoassay (EIA) Kit (Takara, cat# MK101) following the manufacturer’s instructions. The absorbance was measured at 450 nm using a Cytation 5 plate reader (BioTek).

### Statistical analyses

The data were analyzed via Prism 8.0 software (GraphPad). Parametric data were analyzed via one-way analysis of variance (ANOVA) followed by Dunnett’s post hoc test. A significance level of *p* < 0.05 was used to determine statistical significance. The statistical methods applied for each data point are described in the figure legend.

## Electronic supplementary material

Below is the link to the electronic supplementary material.

Supplementary Material 1

Supplementary Material 2

Supplementary Material 3

## Data Availability

The microarray data generated during this study have been deposited in the NCBI Gene Expression Omnibus (GEO) under accession number GSE270805 (https://www.ncbi.nlm.nih.gov/geo/). Proteomic datasets, including raw and processed files, are available within Supplementary Material. For additional information or data requests, please contact J.P. at joonghoon@snu.ac.kr.
