# Supplementary material for: Comparative and pharmacological investigation of bEVs from eight Lactobacillales strains
Source: Sci Rep. 2025 Jul 26;15:27263. doi: 10.1038/s41598-025-12873-z (PMC12297607; doi:10.1038/s41598-025-12873-z)

SUPPLEMENTARY INFORMATION

Supplementary Tables

Supplementary Table 1. KEGG pathway analysis of bEV-induced DEGs

Supplementary Table 2. Connectivity mapping of bEV to approved drugs

Supplementary Table 3. Proteomic analysis of bEV proteins

Supplementary Figure Legends

Supplementary Figure 1. bEV protein profiles

A. Uncropped bEV protein profiles by Coomassie blue staining. Bacterial strains A to D are unclosed as strains unrelated to this study. **B**. Uncropped Western blotting of LTA images for Fig. 1E. Cropped area is indicated by a red dashed line. M: molecular weight marker, E: bEV, C: cell lysate.

Supplementary Figure 2. bEV cytotoxicity

A. HEK293T cell viability. **B**. KEL FIB cell viability. **C**. LX-2 cell viability. The graph is represented with x-axis as bEV concentration (particles/cell) and y-axis as relative cell viability (%). *$p < 0.05$, **$p < 0.01$, ***$p < 0.001$ vs. vehicle control, one-way ANOVA followed by Dunnett's test.

Supplementary Figure 3. Phylogenetic tree of bacteria strains

The graph and table represent the phylogenetic tree between bacterial strains and their source of isolation.

Supplementary Figure 4. Full-length Western blot images of key proteins involved in collagen synthesis in NIH3T3 cells

A. Control (Ctrl), TGF-β, and EVs from *L. rhamnosus* and *L. fermentum*-treated groups. **B**. Ctrl, TGF-β, and EVs from *L. acidophilus*, and *S. thermophilus*-treated groups. All samples were analyzed in triplicate. Cropped areas are indicated by red dashed lines.

Supplementary Figure 5. Full-length Western blot images of key proteins involved in collagen synthesis in LX-2 cells

A. Control (Ctrl), TGF-β, and EVs from *L. rhamnosus* and *L. fermentum*-treated groups. **B**. Ctrl, TGF-β, and EVs from *L. acidophilus*, and *S. thermophilus*-treated groups. All samples were analyzed in triplicate. Cropped areas are indicated by red dashed lines.

Supplementary material. bEV proteome FASTA files by strain

Lacticaseibacillus paracasei (sp_incl_isoforms TaxID=1597_and_subtaxonomies).fasta

Lacticaseibacillus rhamnosus (sp_incl_isoforms TaxID=47715_and_subtaxonomies).fasta

Lactiplantibacillus plantarum (sp_incl_isoforms TaxID=1590_and_subtaxonomies).fasta

Lactobacillus acidophilus (sp_incl_isoforms TaxID=1579_and_subtaxonomies).fasta

Lactococcus lactis (sp_incl_isoforms TaxID=1358_and_subtaxonomies).fasta

Ligilactobacillus salivarius (sp_incl_isoforms TaxID=1624_and_subtaxonomies).fasta

Limosilactobacillus fermentum (sp_incl_isoforms TaxID=1613_and_subtaxonomies).fasta

Streptococcus thermophilus (sp_incl_isoforms TaxID=1308_and_subtaxonomies).fasta

Supplementary Figure 1A

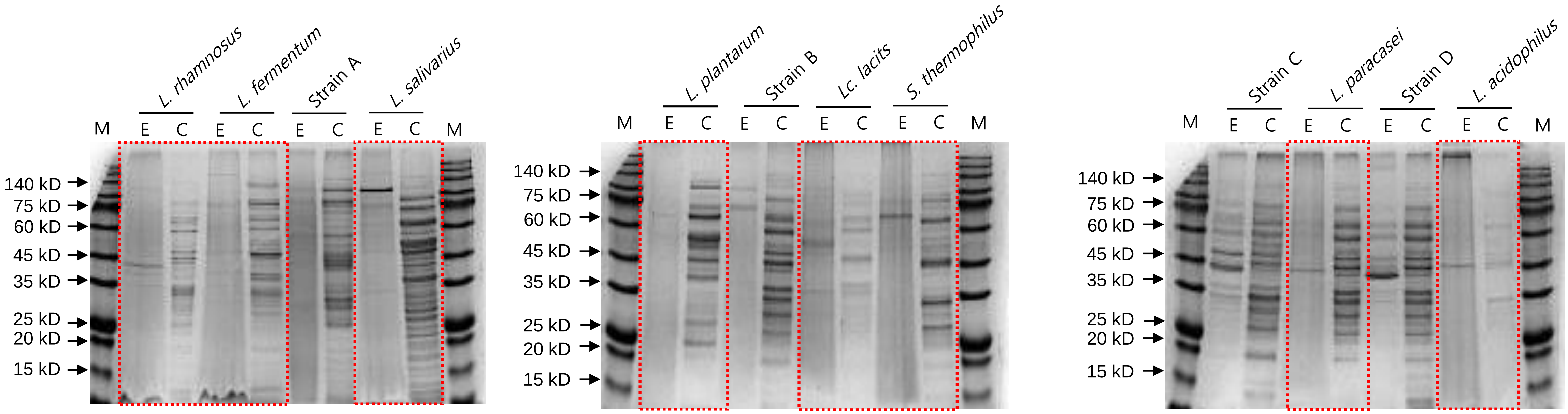

Supplementary Figure 1B

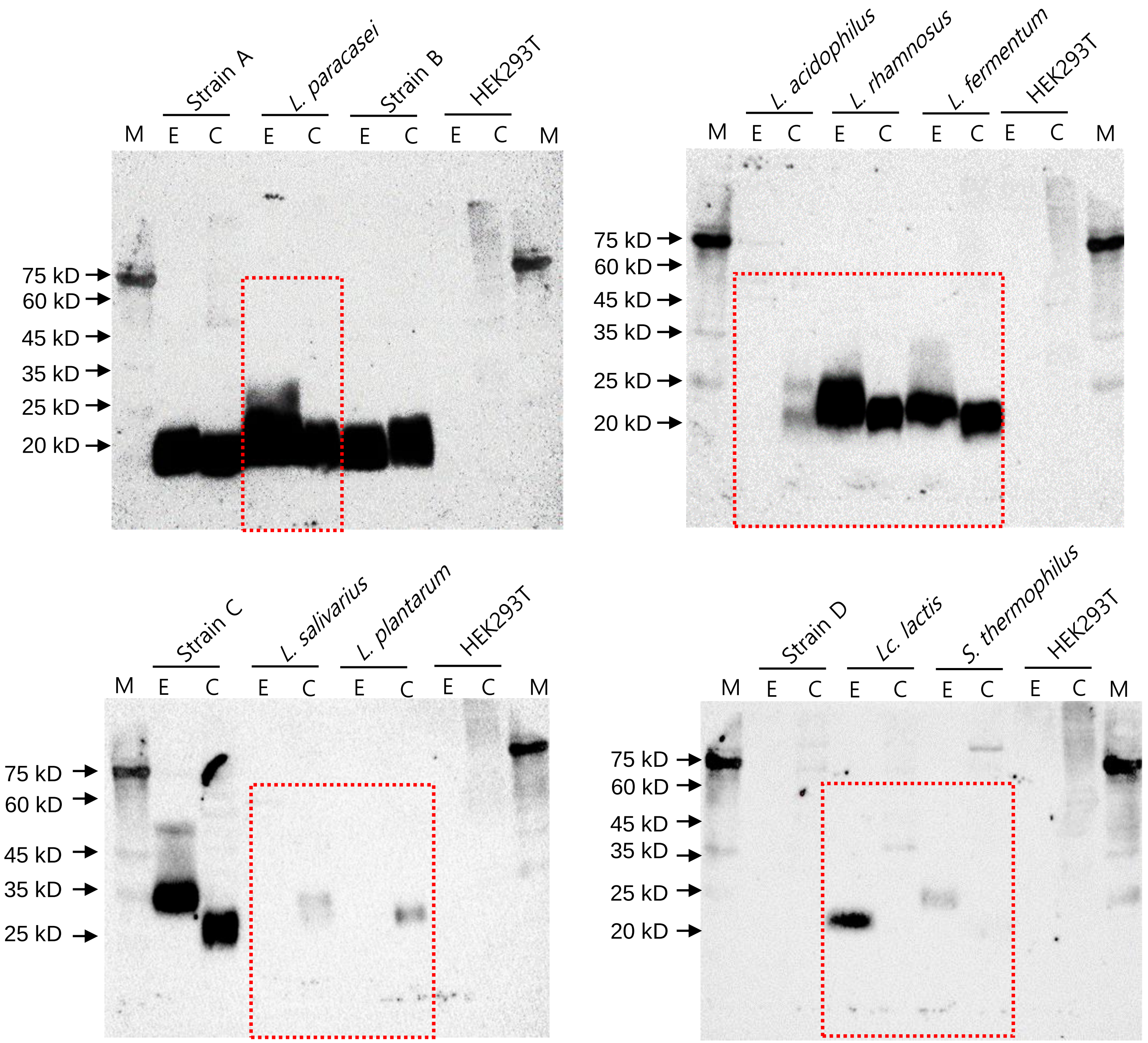

Supplementary Figure 2A

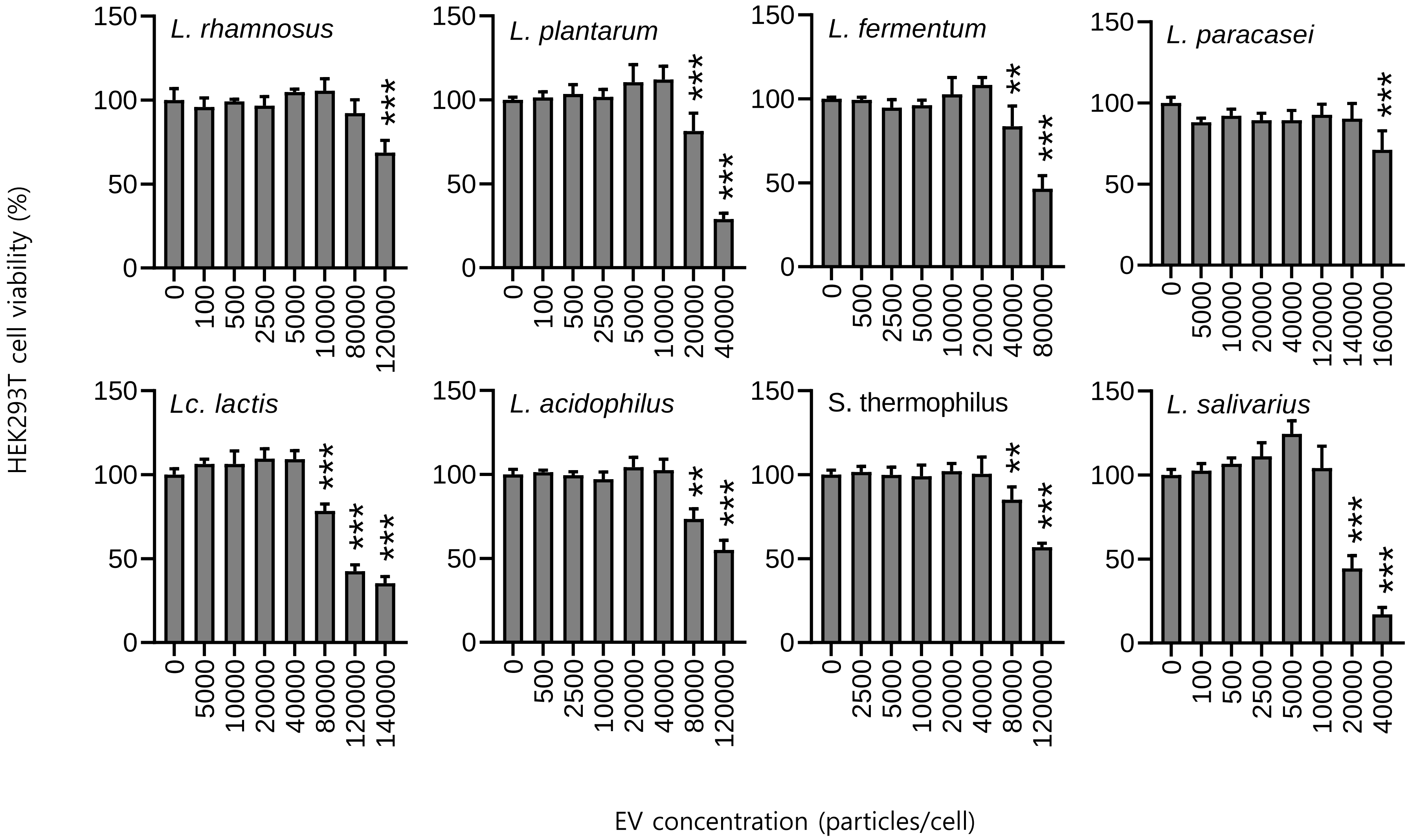

Supplementary Figure 2B

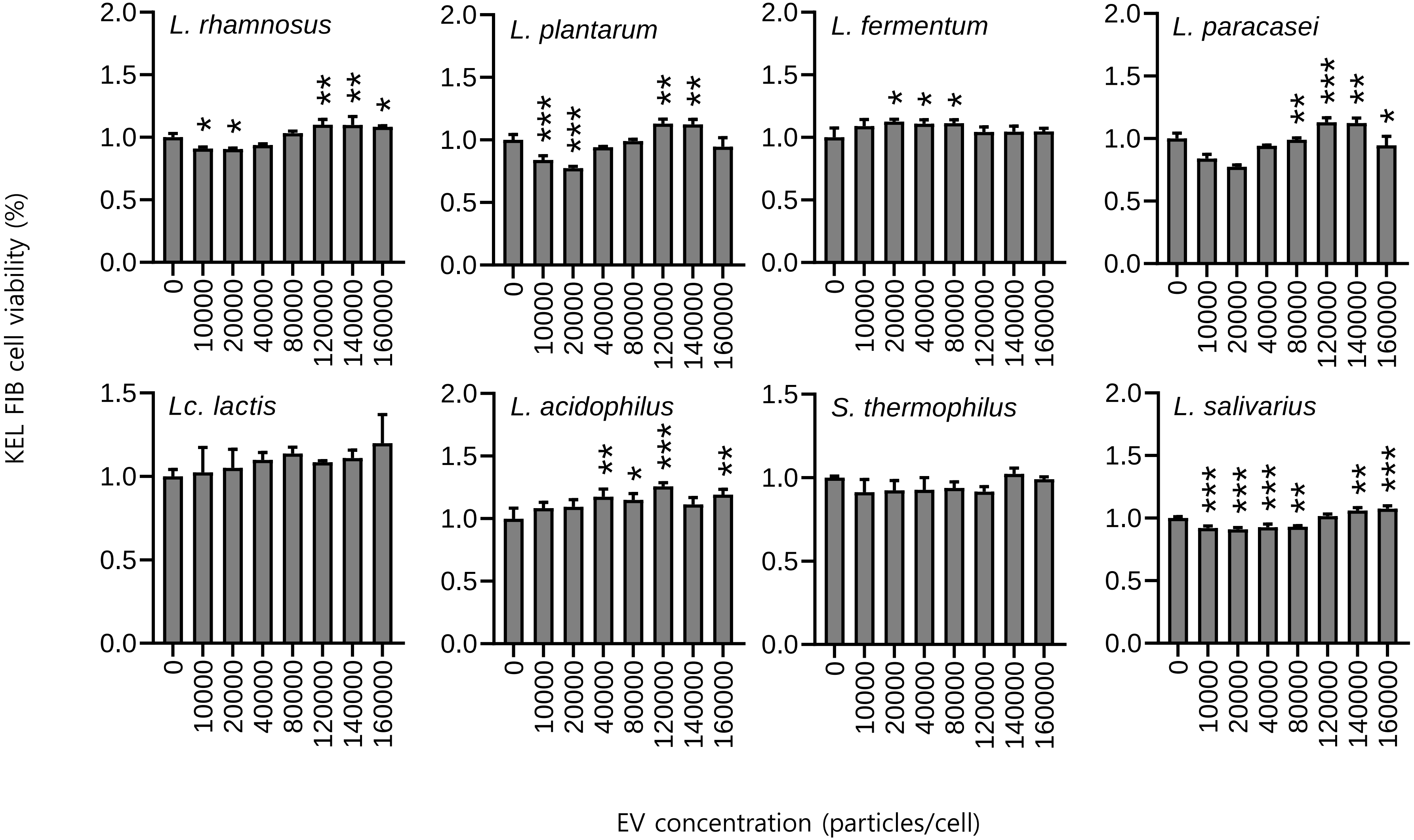

Supplementary Figure 2C

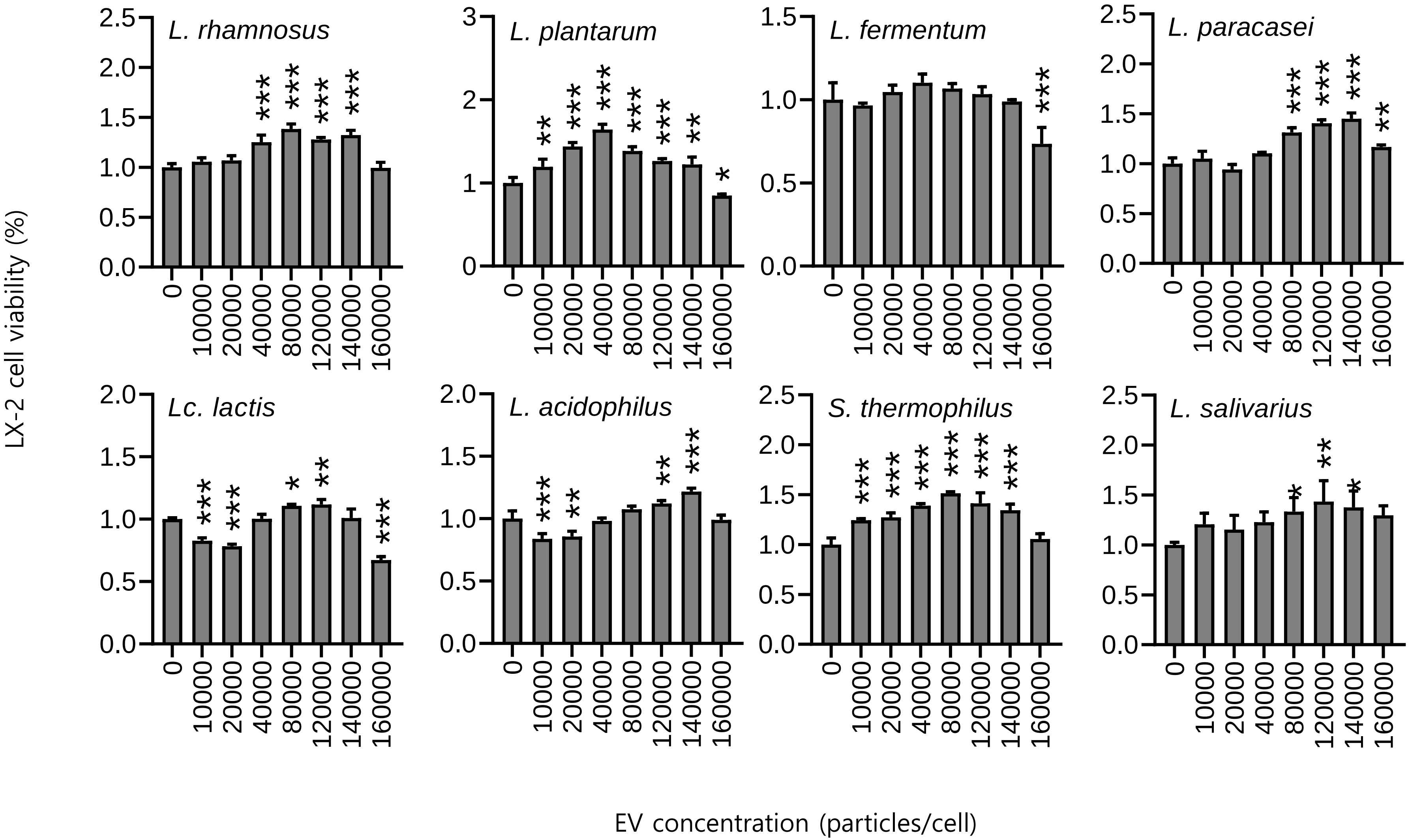

Supplementary Figure 3

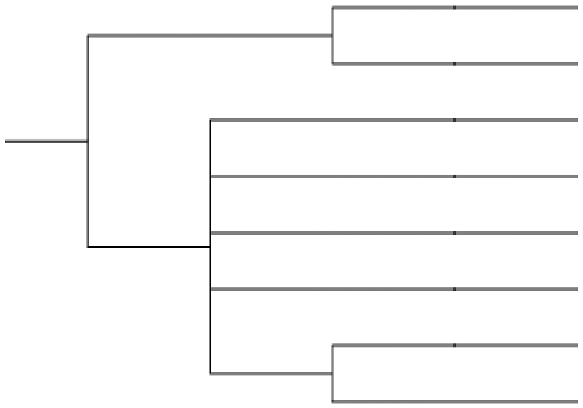

Bacteria strain	Source
S. thermophilus	*Salvia splendens*
Lc. lactis	Soy milk yogurt
L. fermentum	*Citrus Beni Madonna*
L. salivarius	*Salvia splendens*
L. plantarum	Shine Muscat
L. acidophilus	*Salvia splendens*
L. paracasei	Cheese
L. rhamnosus	Milk yogurt

Supplementary Figure 4A

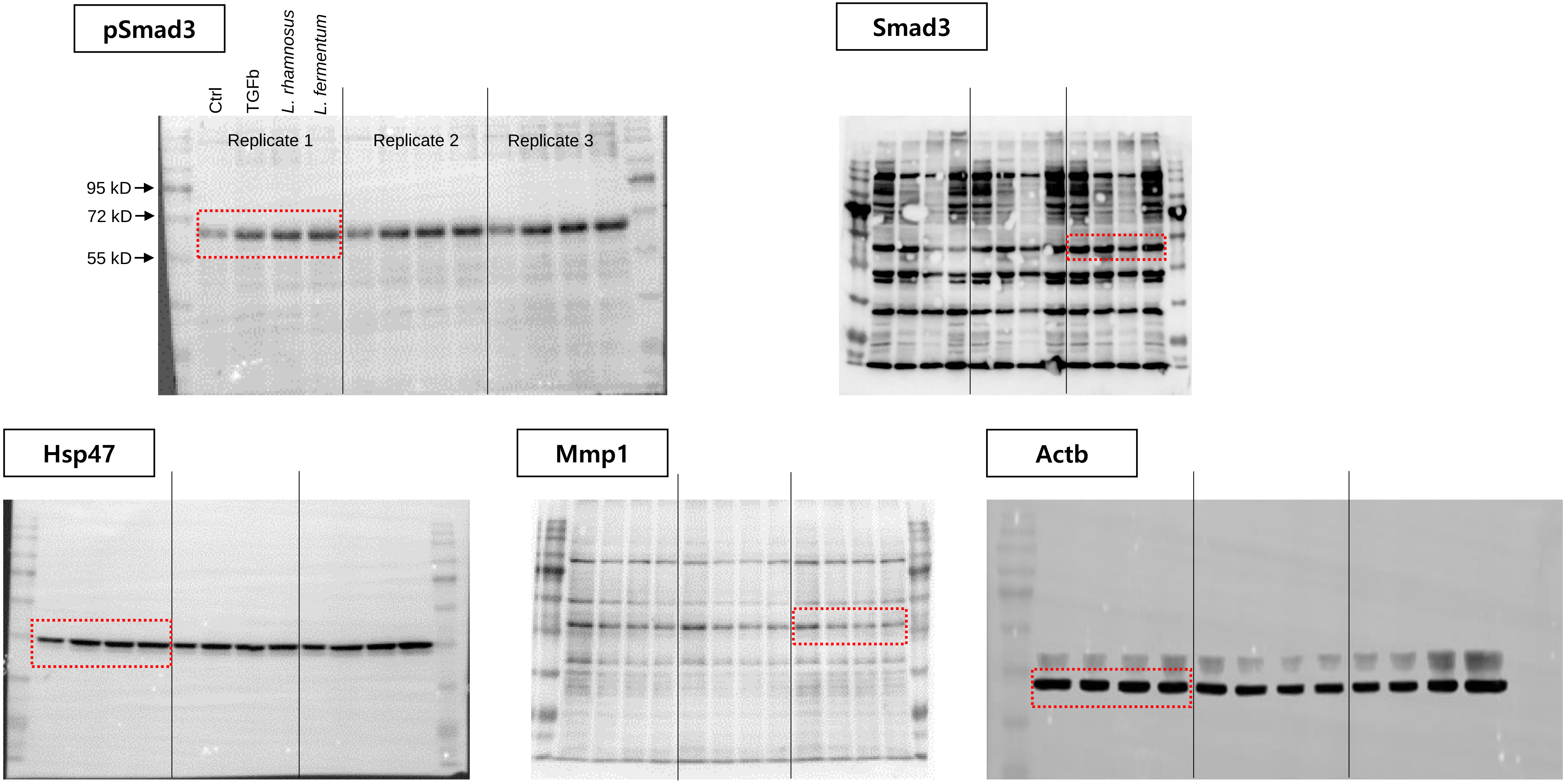

Supplementary Figure 4B

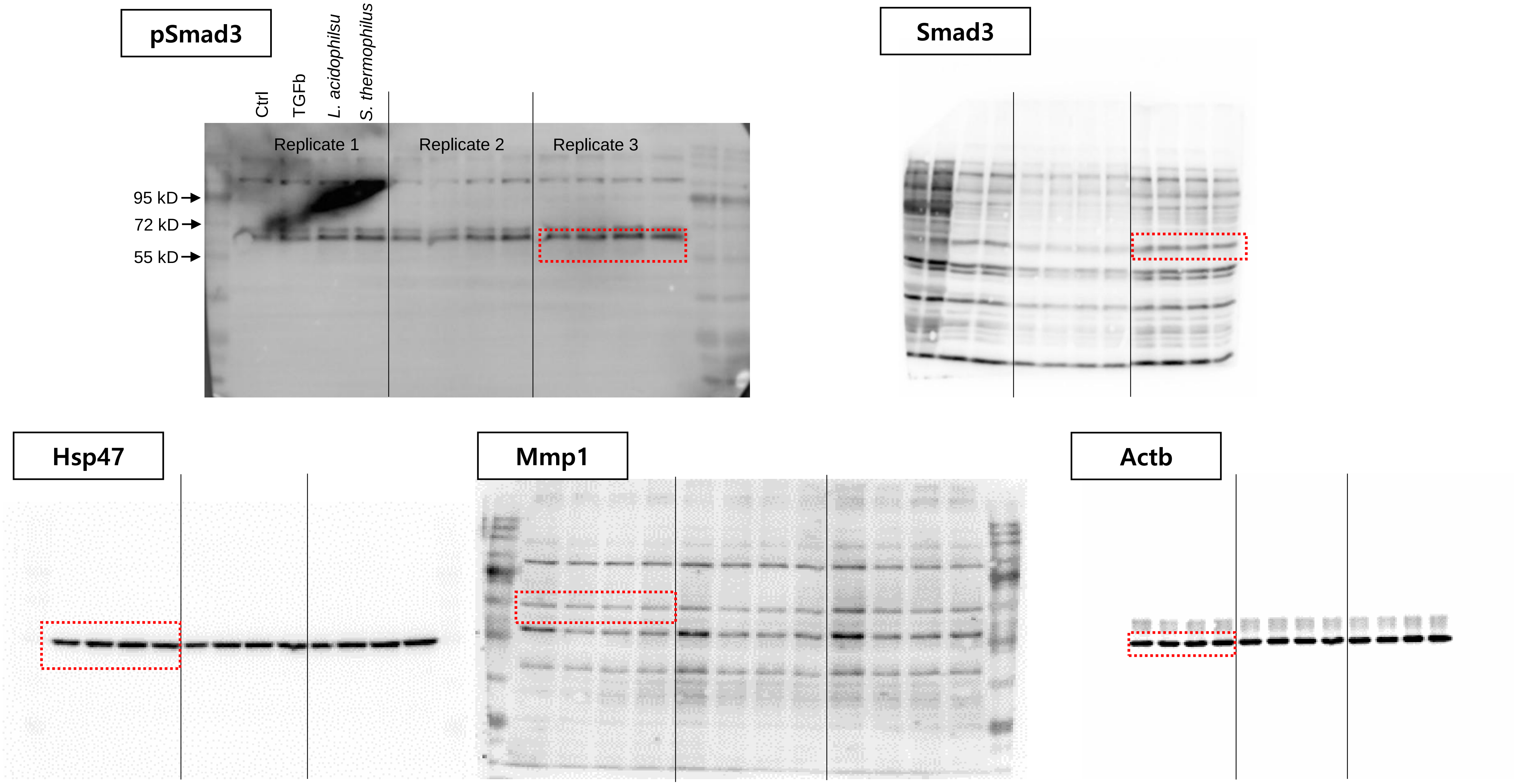

Supplementary Figure 5A

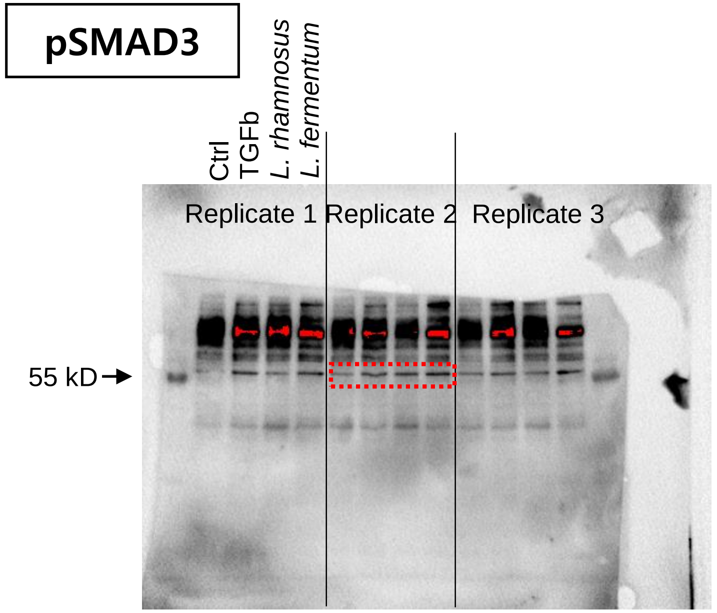

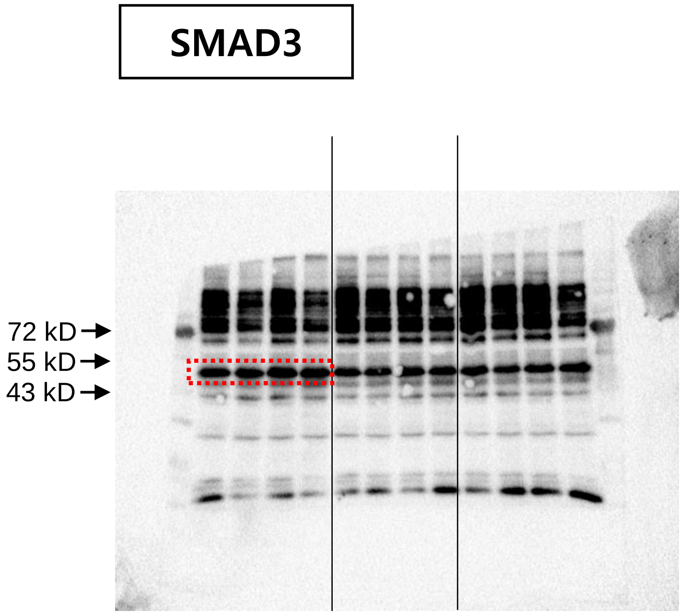

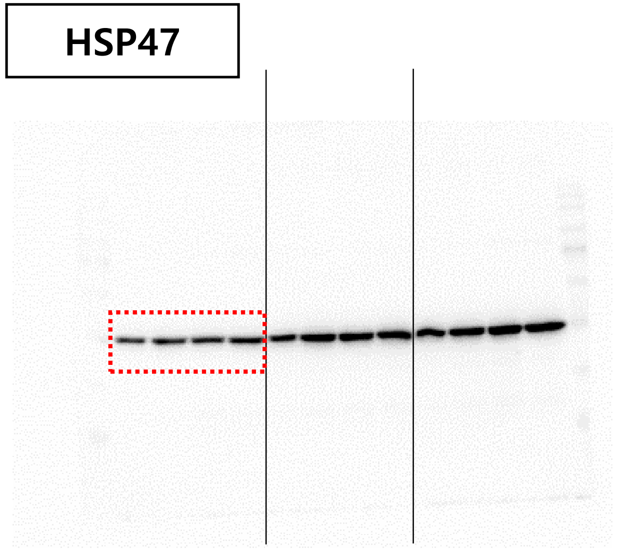

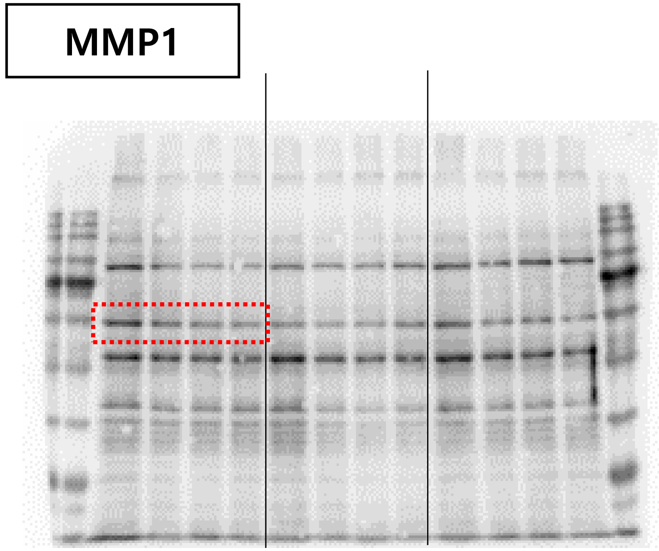

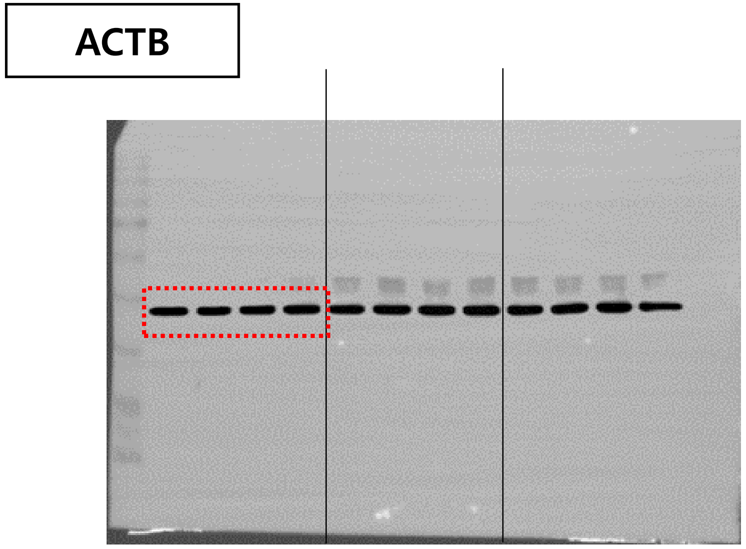

Supplementary Figure 5B

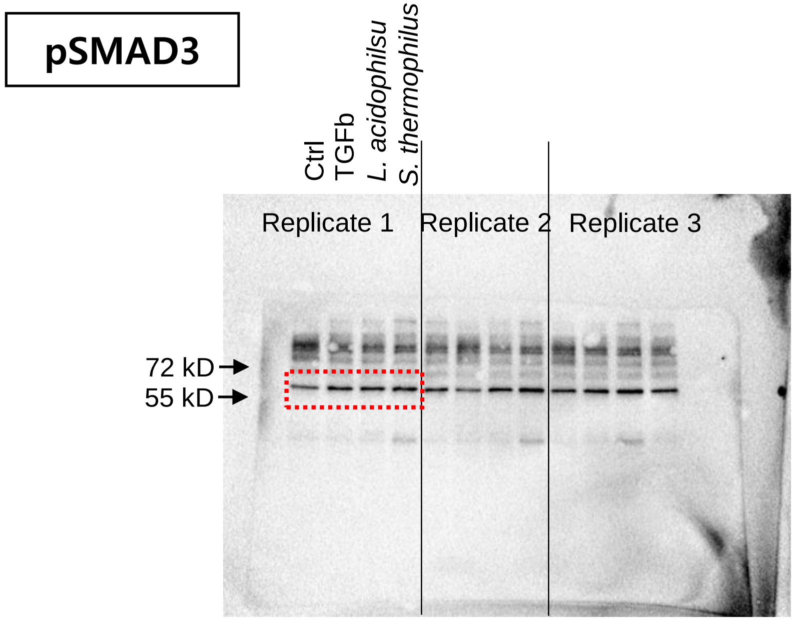

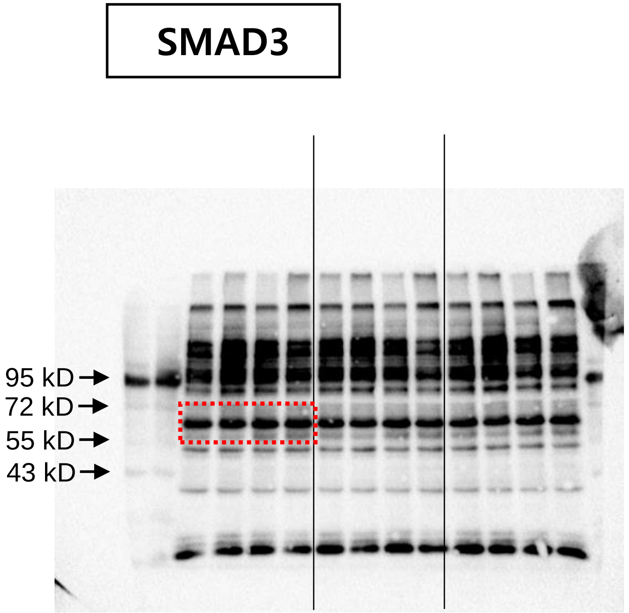

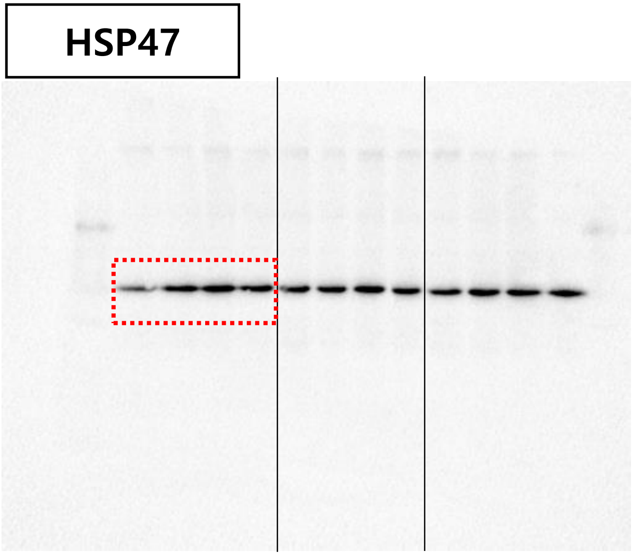

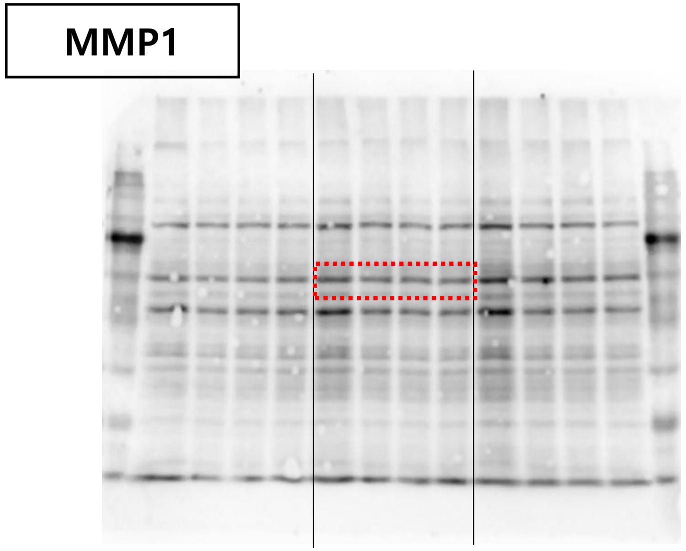

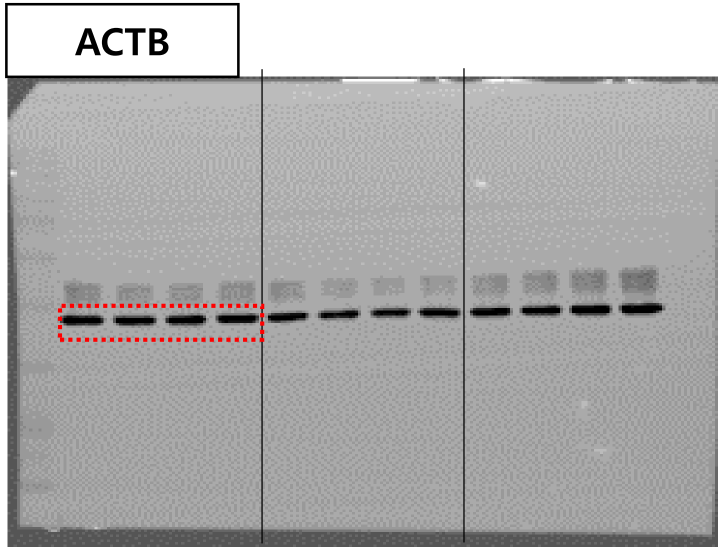

Supplement: Supplementary file 3 — Supplementary Material 3 [file 41598_2025_12873_MOESM3_ESM.pdf]
